# Rumen-protected methionine supplementation alters lipid profile of preimplantation embryo and endometrial tissue of Holstein cows

**DOI:** 10.3389/fvets.2023.1301986

**Published:** 2024-01-17

**Authors:** Stephanie L. Stella, Anne R. Guadagnin, Diego A. Velasco-Acosta, Christina R. Ferreira, Marcello Rubessa, Matthew B. Wheeler, Daniel Luchini, Felipe C. Cardoso

**Affiliations:** ^1^Department of Animal Sciences, University of Illinois, Urbana, IL, United States; ^2^Schothorst Feed Research, Lelystad, Netherlands; ^3^The Colombian Corporation for Agricultural Research (CORPOICA), Bogotá, Colombia; ^4^Metabolite Profiling Facility, Bindley Bioscience Center, Purdue University, West Lafayette, IN, United States; ^5^Adisseo NA, Alpharetta, GA, United States

**Keywords:** amino acids, lipid profiling, embryo, endometrium, dairy cow

## Abstract

Our objective is to evaluate the effects of feeding rumen-protected Met (RPM) throughout the transition period and early lactation on the lipid profile of the preimplantation embryos and the endometrial tissue of Holstein cows. Treatments consisted of feeding a total mixed ration with top-dressed RPM (Smartamine^®^ M, Adisseo, Alpharetta, GA, United States; MET; *n* = 11; RPM at a rate of 0.08% of DM: Lys:Met = 2.8:1) or not (CON; *n* = 9, Lys:Met = 3.5:1). Endometrial biopsies were performed at 15, 30, and 73 days in milk (DIM). Prior to the endometrial biopsy at 73 DIM, preimplantation embryos were harvested via flushing. Endometrial lipid profiles were analyzed using multiple reaction monitoring-profiling and lipid profiles of embryos were acquired using matrix assisted laser desorption/ionization mass spectrometry. Relative intensities levels were used for principal component analysis. Embryos from cows in MET had greater concentration of polyunsaturated lipids than embryos from cows in CON. The endometrial tissue samples from cows in MET had lesser concentrations of unsaturated and monounsaturated lipids at 15 DIM, and greater concentration of saturated, unsaturated (specifically diacylglycerol), and monounsaturated (primarily ceramides) lipids at 30 DIM than the endometrial tissue samples from cows in CON. In conclusion, feeding RPM during the transition period and early lactation altered specific lipid classes and lipid unsaturation level of preimplantation embryos and endometrial tissue.

## Introduction

Fertility is the main physiological process that is affected by dietary imbalances (1). During the transition period, high-producing dairy cows face increased nutritional requirements to support colostrum and milk production while voluntary feed intake is limited, thus experiencing at least some degree of negative energy and protein balance (2–4). This nutritional imbalance during the transition period increases the risk for metabolic disorders, including reproductive tract inflammatory disorders, which can impair the fertility of dairy cows (5, 6). Multiple studies have established the importance of implementing nutritional strategies to improve fertility in dairy cows (5, 7–9). Unbalanced maternal nutrition also impacts embryonic development (10), especially during early stages of development, when embryos require several indispensable nutrients to support the rapid cell division (11). In addition, low-intake of Met, choline, or folate can decrease the concentration of S-adenosylmethionine (SAM), potentially leading to hypomethylation of the DNA (12), and thus altering gene expression and phenotype (13). For instance, reductions in Met and B vitamins resulted in altered epigenetics and DNA methylation of offspring, leading to changes in health-related phenotypes in sheep (14). Therefore, Met assumes particular significance during the developmental stage of the embryo. Methionine is an important methyl donor (15), and is a key player in the one-carbon catabolism, as noted in studies by Locasale (16) and Shiraki et al. (17). This process not only supplies the essential building blocks required for biosynthesis but also generates antioxidant components through the transsulfuration pathway and intermediate metabolites that serve as substrates or cofactors for various enzymatic activities, as elucidated by Xu and Sinclair (18) and summarized in Coleman et al. (19). Supplementation of rumen-protected Met (RPM) guarantees the intestinal delivery of this limiting amino acid (20), increasing circulating plasma Met for utilization (21, 22).

*In vitro* studies report that the amino acid availability in culture media affects the embryo’s development (23–25). Bonilla et al. (23) reported that the Met requirements for the preimplantation embryo is between 14 and 21 μM/L. Additionally, there was a lower percentage of oocytes that developed to blastocyst in cultures without Met at day 7 and 8, and the proportion of expanded, hatching, or hatched blastocysts on day 7 was reduced at lower concentrations of Met (23). Although less available, *in vivo* studies reported that increased supply of intestinally available Met to dairy cows during the transition period alters the transcriptome of bovine blastocyst, which could impact embryonic and adult functions (26, 27). Additionally, supplementation of RPM increased the expression of 3β-HSD in follicular cells, which aids in synthesis of all classes of steroid hormones that are involved in pregnancy maintenance processes (28). In the same study, Acosta et al. (28) also reported increased concentration of Met in the follicular fluid harvested from cows that were supplemented with RPM. While the direct effect of Met on the development of the preimplantation bovine embryo has not yet been investigated, the addition of 20 amino acids in media resulted in an increased number of cells in the blastocyst, suggesting improved growth (29).

Despite some differences in the response according to the organ of interest, Met restriction has been associated with altered lipid metabolism, particularly in monogastric studies (30). Thus, an increase in Met availability could also impact lipid metabolism. For instance, Obeid et al. (15) reported that Met aids in the synthesis of phospholipids. Additionally, Met can act as a lipotropic agent, assisting with lipid removal from the liver through stimulation of very low-density lipid formation (27). Furthermore, Acosta et al. (31) reported an increased lipid accumulation in preimplantation embryos from cows fed RPM during the transition period, suggestive of a response to increased fatty acid concentration in blood because Met stimulates triacylglycerol (TG) clearance from the liver (31). Therefore, dietary supplementation of Met could possibly increase the lipid availability in the embryo.

Waladkhani et al. (32) reported an increase of arachidonic acid and docosahexaenoic acid in rats upon supplementation with Met, suggesting that Met could be increasing the activity of desaturases. In support, the enzyme stearoyl-CoA desaturase-1 appears to be directly related to the metabolism of sulfur amino acids, particularly cysteine (33). Cysteine is a product of the Met metabolism (34). Additionally, Met-restricted diets resulted in a decrease in SCD-1 expression by the liver in mice (35). Thus, we hypothesize that increased availability of Met during the transition period and early lactation would affect lipid diversity and accumulation in the bovine uterus and embryo, possibly because of an increase in desaturases. Additionally, there is limited research regarding the effects of RPM on the lipid profile of bovine embryos and on the extent of lipid classification in the bovine uterus. Therefore, we aim to investigate the lipid profile in the preimplantation embryo and in the endometrial tissue of Holstein cows fed RPM during the transition period and early lactation.

## Materials and methods

### Experimental design and dietary treatments

All experimental procedures were approved by the University of Illinois (Urbana-Champaign) Institutional Animal Care and Use Committee (no. 13023). Detailed procedure is described elsewhere (31). Briefly, 20 Holstein cows entering their second or greater lactation (parity 3.0 ± 1.2) enrolled in the experiment at 21 ± 1 days before expected calving date. Cows were randomly assigned to one of two treatments, which consisted either of a TMR top-dressed with RPM [MET; *n* = 11; RPM at a rate of 0.08% of dry matter (DM): Lys:Met 2.8:1 (Smartamine^®^ M, Adisseo, Alpharetta, GA, United States)] or without (CON, *n* = 9; Lys:Met 3.5:1), which were fed until 73 ± 1 days in milk (DIM). Cows were blocked according to the expected calving date, and within each block, cows were balanced for parity, previous lactation milk yield, and BCS prior the close-up. We included this information in the text now. All cows were milked three times a day and received a complete total mixed ration (TMR) diet that met or exceeded their energy requirements according to the NRC (20). Supplementation of RPM (0.08% of DM) was established based on Zhou et al. (22) and calculated using data of TMR offered on a DM basis.

### Estrus synchronization, superstimulation, and embryo flushing protocol

Protocols for synchronization, super stimulation, and embryo flushing were performed according to Acosta et al. (31). Pre-synchronization protocol consisted of with one injection of prostaglandin F2α (25 mg, intramuscular of dinoprost tromethamine; 5 mL of Lutalyse, Zoetis Animal Health, NJ, United States), at 30 ± 1 DIM, an injection of GnRH (100 μg, intramuscular, of gonadorelin hydrochloride; 2 mL of Factrel, Zoetis Animal Health) at 44 DIM immediately followed by the insertion of a controlled internal drug release (CIDR; Eazi-Breed CIDR, Zoetis Animal Health) containing 1.38 g of progesterone. At 51 Dim, the CIDR was removed and an injection of prostaglandin F2α was administered. At 53 DIM, cows received the second injection of GnRH. At 60 DIM, all follicles greater than 5 mm were aspirated using an ultrasound-guided transvaginal approach, with an 18-gauge × 55 cm aspiration needle. After follicular aspiration, a CIDR device was inserted in all cows. One day and a half later, superstimulation protocol was initiated, with intramuscular administration of FSH equivalent NIHFSH-P1 (400 mg, Folltropin-V, Bioniche, Life Science, Belleville, ON, Canada). Eight subsequent decreasing dosages of FSH were administered at 12-h interval over a period of 4 days. Additionally, all cows received an injection of PGF2α at 63 and 64 DIM, and the CIDR was removed at 65 DIM. Ovulation was induced with administration of GnRH 24 h after CIDR removal. Artificial insemination was performed by the same technician at 12 and 24 h after administration of GnRH, with semen from a commercially available Holstein sire (014HO05388; two doses per insemination). Superstimulation response was assessed by counting the number of CL in each ovary through ultrasound imaging (Ibex Pro portable ultrasound, E. I. Medical Imaging, Loveland, Colorado, United States) and was reported by Acosta et al. (31). The number of CL for cows that received MET (11.2 ± 0.9) was greater (*p* = 0.01) than for cows in CON (9.2 ± 0.9). Finally, embryos were flushed 6.5 days after the first timed artificial insemination and evaluated according to the manual of the International Embryo Transfer Society (36). Embryos were exposed to 1.5 M EG cyroprotectant solution (Vigro Ethylene Gycol^®^, Bioniche Animal Health, Pullman, WA, United States) for 5 min and loaded into 0.25 mL plastic straws before being placed into a temperature controller (Freeze Control^®^, Margaret River, Western, Australia) at – 6.5°C. Afterwards, the straws were seeded, equilibrated for 10 min at – 6.5°C, and cooled at – 0.6°C/min until the temperature reached – 35°C. Recovery rate, number of embryos recovered, embryo quality, and embryo stage were not different between treatments [*p* = 0.22; (31)]. Embryos were stored in liquid nitrogen until analysis.

### Lipid analysis of embryos by MALDI-TOF MS

Embryos from three cows (MET = 1 cow yielding 5 embryos, CON = 2 cows yielding 3 embryos total) that were quality = 1 and were at stage = 4 or greater were used for lipid analysis (36). Each embryo was washed in phosphate buffer solution (PBS) three times to remove media and 25% methanol two times to remove salts that could disturb ionization. Using a mouth pipette, the embryos were placed onto an Opti-TOF 384 well plate (Ab SCiex, Concord, Ontario, CA), and allowed to dry before being stored at – 80°C. Individual embryos had their lipid profile acquired via matrix-assisted laser desorption/ionization time-of-flight mass spectrometry (MALDI-TOF MS) at the Bindley Bioscience Center at the Discovery Park of Purdue University (West Lafayette, IN). Prior to analysis, a matrix was administered to the sample plate, which consisted of 8 mL of TA30 solvent (30:70 [v/v] acetonitrile:0.1% TFA in water) to 160 mg of 2,5-DHB (2,5–Dihydroxybenzoic acid) for a total volume of 8 mL of matrix solvent that was loaded and administered 16 times in a cross section pattern at 45°C by a matrix sprayer (HTX TM-Sprayer, HTX Technologies, Chapel Hill, NC, United States). From there, images and mass spectra were obtained in a MALDI 4800 TOF instrument (AB SCiex, Concord, Ontario, CA) while utilizing the 4,000 Series Explorer Software (v3.7, AB SCIEX) in reflectron at the positive ion mode. All images were acquired with the 4,800 Imaging Tool Software^1^ (M. Stoeckli, Novartis Pharma, Basel, Switzerland) with an imaging raster of 100 μm at 50 shots per subspectrum in the m/z range of 700 to 1,200.

### Uterine biopsy collection

Uterine biopsy was performed at 15, 30, and 73 DIM from all eligible cows. Exclusion criteria for the performance of uterine biopsy were presence of vaginal discharge (Metricheck) score plus smell >3 on the biopsy day or a drop in DMI greater than 20 kg from the day before. Uterine biopsies were not performed on 10 cows (CON = 4, MET = 6) on d 15; on 11 cows (CON = 5, MET = 6) on d 30; and on 7 cows on d 73 (CON = 4, MET = 3). Endometrial tissue samples were collected transcervically from the body of the uterus using a biopsy forceps (48 cm in length, 2 cm diameter; Aries Surgical, Davis, CA, United States). Endometrial tissue was collected at approximated 2 cm beyond the end of the cervix. Endometrial samples were then placed into sterile DNA/RNA-free cryotubes (Simport, Beloeil, Quebec, Canada), and flash-frozen in liquid nitrogen.

### Lipid analysis by MRM-profiling

Lipid analysis on uterine tissue samples were performed at the Bindley Bioscience Center using Multiple Reaction Monitoring profiling (MRM-profiling) mass spectrometry. The uterine samples were homogenized, and the lipids were extracted before the discovery phase of analysis using the Bligh and Dyer method (37). The discovery phase consisted of pooling the lipid extracts from each sample, resulting in two pooled samples that were representative of each treatment, to identify the groups of lipids that were present in the samples. Methods used for monitoring the lipids found in the discovery phase are in Table 1. From there, the samples were delivered through a micro-autosampler (G1377A) into a triple quadrupole (QqQ) mass spectrometer (6,460, Agilent Technologies, San Jose, CA). Selection of the parent ion occurred at the first quadrupole, collision induced dissociation caused fragmentation at the second quadrupole, and finally, the fragment was monitored at the third quadrupole. Each sample was run through five different methods (4 methods for positive and 1 for negative ion mode) during the screening step to ensure accurate screening of the targeted lipids found during the discovery phase. Solvent (ACN + 0.1% formic acid) was pumped through the mass spectrometer between each injection of the sample. Further details regarding the MRM-profiling method have been described (38, 39).

**Table 1:** Discovery phase methods and respective scan modes for individual screening of uterine biopsy samples for 15, 30 and 73 DIM.

Method	Ion mode	Number of transitions	Scan mode used for the discovery step
1	Positive	178	Phosphatidylcholine (PC), Sphyngomyelin (SM), Glycerolipids containing palmitoleic acid (16:1), palmitic acid (16:0), DHA (22:6), tetracosahexaenoic acid (24:6) and dotriacontanoic acid (32:0).
2	Positive	178	Glycerolipids containing palmitic acid (16:0), linolenic acid (18:3), linoleic acid (18:2), oleic acid (18:1), stearic acid (18:0), eicosatetraenoic acid (20:4), eicosatrienoic acid (20:3), eicosadienoic acid (20:2), arachidic acid (20:0), docosatetraenoic acid (22:4), docosahexaenoic acid (22:6).
3	Positive	178	Glycerolipids containing 22:6, 22:4, docosadienoic acid (22:2), docosenoic acid (22:1), behenic acid (22:0), lignoceric acid (24:0), Phosphatidylcholine (PC) and Sphyngomyelin (SM).
4	Positive	181	Phosphatidylcholine (PC) and Sphyngomyelin (SM), Ceramides, Phosphatidylinositol (PI), Acylcarnitines, nervonic acid (24:1), montanic acid (28:0).
5	Negative	64	Glycerolipids containing 14:1 residue, 16:0, 18:0, Phosphatidylinositol (PI).

Less than 200 MRMs were used per method due to limited time for data collection.

### Statistical analyses

Statistical analyses were performed using Metaboanalyst 3.0^2^ and SAS 9.4 (SAS Institute Inc. Cary, NC, United States). Lipid profiles were processed as relative ion count intensities (each ion in the mass spectrum had the ion counts divided by the total ion count of all ions in the mass spectrum) and 10% of background noise in relation to the highest peak value was removed for the embryo data to reduce false positives. Principal component analysis (PCA) was performed by replacing values below the 10% threshold with means and auto scaling according to the Metaboanalyst (see Footnote 2). Only auto scaling was applied for the uterine biopsy data due to background noise being removed during the discovery phase of analysis. All PC with eigenvalues (λ) greater than or equal to 0.9 were extracted, and only loadings greater than 0.49 were discussed. Lipids were tentatively attributed based on the LipidMaps database^3^ considering a mass tolerance appropriated for the instruments used (0.4 Da for MALDI and up to unitary resolution for the QqQ). For uterine biopsies, lipid attribution was also pursued using METLIN.^4^ For MALDI-MS data, a standard deviation of 0.4 Da was used for the theoretical masses m/z 708.90 and 732.90 due to limited attributions from the database. Additionally, the theoretical mass of m/z 710.90 was used to attribute the lipids for m/z 708.90 due to one unsaturation loss equaling 2 mass units for the embryo data. Based on previous experience using MALDI and 2,5-dihydroxybenzoic acid as a matrix, as well as MS/MS of lipids that are present in oocytes, we only considered phosphatidylcholine (PC), sphingomyelin (SM), and triacylglycerol (TG) as possible lipids for the tentative attributions in the embryos. Finally, variables that were considered to be important according to the PCA (i.e., loadings in the top 5%) were analyzed using the MIXED procedure of SAS. The model for the previously stated response variables contained the fixed effect of treatment. Cow was considered the experimental unit and treated as a random effect. Residual distribution was evaluated for normality and homoscedasticity of variance in all analyses. Statistical significance was declared as *p* ≤ 0.05 and tendency at 0.05 > *p* ≤ 0.15.

## Results

Productive responses and uterine health status data are reported in Zhou et al. (22) and (61), respectively. Briefly, cows fed RPM had greater DMI during close-up and first 30 DIM, greater milk yield, energy-corrected milk, and fat-corrected milk (22). In addition, the incidence of ketosis and retained placenta tended to be lower in cows that received RPM (22). Body weight and energy balance did not differ between cows that were fed RPM or not (22). Cows fed RPM had a greater percentage of uterine polymorphonuclear (PMN) cells at 15 DIM, but lower at 30 DIM and at 73 DIM, than cows in CON (61).

### Embryo lipid analysis

To better explain which lipids were detected in the embryos, four PCs were extracted from the PCA. These four PCs accounted for 85% of the total variability (Table 2). Principal component 2 represents embryos that mainly have phosphatidylcholine (PC) lipids. Principal component 3 represents embryos that mainly have TG lipids, whereas PC4 is an even mixture of TG, sphingomyelin (SM), and PC lipids. Analysis of the relative amounts of lipids that were of interest based on PCA for embryos retrieved at 73 DIM is in Table 3. The embryos from cows in MET tended (*p* = 0.10) to have lower monounsaturated lipid amounts than cows in CON. Alternatively, the embryos from cows in MET had greater (*p* < 0.01) polyunsaturated lipid amounts, specifically TG and PC lipids, compared to the embryos from cows in CON. Tentative attributions for lipids of interest for embryos based on PCA are presented in Supplementary Table S1. The extracted loadings (PC1: loadings ≥0.15459; PC2: loadings ≥0.20502; PC3: loadings ≥0.19442; PC4: loadings ≥0.23844) indicate that PC1 represents animals that have triglycerides lipids present. Scores plots for lipid markers in embryos retrieved at 73 DIM are in Supplementary Figure S1.

**Table 2:** Principal components (PC), proportion (p), and cumulative proportion (Cp) of the principal component analysis for embryos retrieved at 73 DIM.

PC	p	Cp
PC1*	37.9	37.9
PC2*	19.8	57.6
PC3*	15.4	73.1
PC4*	11.9	85.0 93.2 97.8
PC5	8.2	93.2
PC6	4.6	97.8

*Extracted principal components.

**Table 3:** Least squares means and associated standard errors of normalized lipid marker intensity ion counts^1^ for embryos from Holstein cows supplemented with rumen-protected Met (MET) or not (CON) from 21 days before calving until 73 DIM.

Variable	Treatment			*p* value
	CON^2^	MET^3^	SEM^4^	
Lipid attribution, *m/z*				
[PC(P-30:2) + Na]^+^, 708.90	2.25	1.79	0.17	0.09
[SM(d34:1) + Na]^+^, 725.51	5.44	2.13	1.30	0.11
[TG(50:4) + H]^+^; [TG(48:1) + Na]^+^, 827.50	1.41	2.78	0.37	0.06
[PC(42:1) + H]^+^; [PC(P-42:4) + Na]^+^, 872.90	1.48	2.10	0.16	0.03
[PC(42:0) + H]^+^; [PC(O-42:4) + Na]^+^, 874.90	5.67	8.30	0.78	0.04
[TG(54:8) + H]^+^; [TG(52:5) + Na]^+^, 875.90	2.07	3.01	0.31	0.05
[TG(54:6) + H]^+^; [TG(52:3) + Na]^+^, 879.50	1.56	4.81	1.26	0.11
non-attributed, 906.70	1.34	2.92	0.63	0.12
[TG(66:12) + H]^+^; [TG(64:9) + Na]^+^; [TG(64:17) + K]^+^; [TG(62:3) + K]^+^, 1,036	1.29	3.27	0.66	0.07
Group				
Unsaturated	21.16	24.25	1.98	0.26
Monounsaturated	8.32	5.05	1.32	0.10
Polyunsaturated	17.07	25.90	1.42	0.003

^1^Intensity ion counts multiplied by 1000. ^2^CON, control, no rumen protected Met, *n* = 3. ^3^MET, rumen protected Met, *n* = 5. ^4^SEM multiplied by 1000.

### Endometrial biopsy lipid analysis at 15 DIM

To better explain which lipids were found in the uterine endometrial biopsies obtained at 15 DIM, method 1 had PCs extracted accounting for 93.4% variability, and methods 2–5 had 2 PCs extracted each accounting for 79.6, 83.9, 85.3, and 91.9% variability, respectively (Table 4). Grouping identification, while an added benefit, was not revealed for PC1 for methods 1, 2, 4, or 5. Analysis of lipid markers that were of interest according to the PCA for 15 DIM are in Table 5. Cows in CON tended (*p* = 0.14) to have higher amounts of saturated lipids compared to cows in MET. Similarly, cows in CON tended (*p* = 0.13) to have higher amounts of unsaturated/monounsaturated lipids, specifically PC lipids, than cows in MET. Tentative attributions for lipids of interest for uterine endometrial biopsies obtained at 15 DIM based on PCA are in Supplementary Table S3. The extracted loadings (Method 1: PC1 loadings ≥0.077435; Method 2: PC1 loadings ≥0.093893 and PC2 loadings ≥0.17326; Method 3: PC1 loadings ≥0.095559 and PC2 loadings ≥0.14156; Method 4: PC1 loadings ≥0.087559 and PC2 loadings ≥0.15073; Method 5: PC1 loadings ≥0.14552 and PC2 loadings ≥0.27031) are in Supplementary Table S4. Scores plots for methods 1–5 for lipid markers in uterine biopsies retrieved at 15 DIM are found in Supplementary Figures S2–S6.

**Table 4:** Principal components (PC) of each method, proportion (p), and cumulative proportion (Cp) of the principal component analysis for uterine biopsies retrieved at 15 DIM.

Variable	p	Cp
Method 1		
PC1*	93.4	93.4
PC2	3.3	96.8
PC3	1.9	98.6
PC4	0.6	99.2
Method 2		
PC1*	63.5	63.5
PC2*	16.1	79.6
PC3	8.9	88.5
PC4	4.8	93.3
Method 3		
PC1*	61.2	61.2
PC2*	22.6	83.9
PC3	5.3	89.1
PC4	4.6	93.8
Method 4		
PC1*	71.4	71.4
PC2*	14.0	85.3
PC3	5.6	91.0
PC4	5.2	96.2
Method 5		
PC1*	71.2	71.2
PC2*	20.7	91.9
PC3	3.6	95.6
PC4	1.9	97.5

*Extracted principal components.

**Table 5:** Least squares means and associated standard errors of normalized lipid marker intensity ion counts^1^ for uterine endometrial biopsies obtained at 15 DIM from Holstein cows supplemented with rumen-protected Met (MET) or not (CON) 21 days before calving until 73 DIM.

	Treatment			*p* value
Variable	CON^2^	MET^3^	SEM^4^	
Method 3, *m/z*				
PC (O-34:1); PC (P-34:0), 746.4→184.2	6.05	4.89	0.52	0.15
PC (O-34:1); PC (P-34:0), 746.5→184.2	4.46	3.61	0.35	0.13
PC (34:1), 760.7→184.2	2.66	2.13	0.23	0.14
Group				
Saturated	10.51	8.50	0.86	0.14
Unsaturated/ Monounsaturated	13.16	10.62	1.07	0.13

^1^Intensity ion counts multiplied by 1000. ^2^CON, control, no rumen protected Met, *n* = 5. ^3^MET, rumen protected Met, *n* = 5. ^4^SEM multiplied by 1000. *Variables for Methods 1, 2, 4, and 5 were not statistically different.

### Endometrial biopsy lipid analysis at 30 DIM

To better explain which lipid markers are present in the endometrial biopsies obtained at 30 DIM, two PCs were extracted for methods 1–5 with the following variability: 97.5, 92.2, 85.5, 85.4, and 85.8%, respectively (Table 6). Analysis of lipids of interest according to the PCA for 30 DIM are in Table 7. Cows in MET tended (*p* = 0.10) to have increased saturated lipid amounts compared to cows in CON. Cows in MET had increased (*p* ≤ 0.05) amounts of unsaturated and monounsaturated lipids, specifically diacylglycerol (DG) and ceramides (CER), in comparison with cows in CON. Tentative attributions for lipids of interest for uterine endometrial biopsies obtained at 30 DIM based on PCA are in Supplementary Table S5. The extracted loadings (Method 1: PC1 loadings ≥0.082637 and PC2 loadings ≥0.15433; Method 2: PC1 loadings ≥0.092384 and PC2 loadings ≥0.14449; Method 3: PC1 loadings ≥0.0884 and PC2 loadings ≥0.15648; Method 4: PC1 loadings ≥0.098898 and PC2 loadings ≥0.12144; Method 5: PC1 loadings ≥0.14766 and PC2 loadings ≥0.27869) are in Supplementary Table S6. Identification of groups based on extracted loadings for methods 1, 2, and 5 did not reveal valuable information due to the vast possibilities of tentative lipids. Scores plots for methods 1–5 for lipid markers in uterine biopsies retrieved at 30 DIM are found in Supplementary Figures S2–S6.

**Table 6:** Principal components (PC) of each method, proportion (p), and cumulative proportion (Cp) of the principal component analysis for uterine biopsies retrieved at 30 DIM.

PC	p	Cp
Method 1		
PC1*	81.2	81.2
PC2*	16.3	97.5
PC3	1.3	98.8
PC4	0.7	99.5
Method 2		
PC1*	66.5	66.5
PC2*	25.7	92.2
PC3	4.0	96.2
PC4	2.3	98.5
Method 3		
PC1*	71.8	71.8
PC2*	13.7	85.5
PC3	7.0	92.4
PC4	3.0	95.4
Method 4		
PC1*	50.8	50.8
PC2*	34.6	85.4
PC3	6.8	92.2
PC4	2.5	94.8
Method 5		
PC1*	71.4	71.4
PC2*	14.4	85.8
PC3	4.6	90.4
PC4	3.9	94.3

*Extracted principal components.

**Table 7:** Least squares means and associated standard errors of normalized lipid marker intensity ion counts^1^ for uterine endometrial biopsies obtained at 30 DIM from Holstein cows supplemented with rumen-protected Met (MET) or not (CON) 21 days before calving until 73 DIM.

	Treatment			*p* value
Lipid, *m/z*	CON^2^	MET^3^	SEM^4^	
Method 1, *m/z*				
LysoPE (24:6); PS (20:0); CER (d34:1), 554.1→280.8	4.65	10.77	2.70	0.14
DG (36:7), 611.7→354.4	0.59	1.49	0.42	0.15
PI (P-20:0), 612→354.7	1.41	19.81	7.86	0.12
non-attributed, 627.9→354.6	4.08	10.14	2.38	0.10
non-attributed, 627.9→370.6	1.87	3.95	0.94	0.14
non-attributed, 630→356.7	2.58	5.66	1.22	0.10
DG (44:4); PA (36:2), 701.9→428.6	1.82	3.76	0.88	0.14
Method 2, *m/z*				
non-attributed, 432.1→106.8	14.35	8.85	2.07	0.09
non-attributed, 432.2→110.9	16.44	10.48	2.65	0.14
DG (36:8), 609.8→280.5	2.00	7.76	2.13	0.08
DG (36:7); PG (24:0), 611.9→282.6	2.29	6.73	1.70	0.09
DG (36:6); PA (30:4), 613→283.7	1.99	3.85	0.70	0.09
Method 4, *m/z*				
CER (d34:1), 538.3→282.2	2.83	6.67	0.97	0.02
CER (d34:1), 538.9→282.2	2.30	8.99	1.87	0.03
CER (d34:1), 539→282.2	2.82	12.77	2.14	0.01
non-attributed, 539.2→282.2	2.69	12.26	2.78	0.04
CER (d34:0), 540.2→282.2	1.14	2.90	0.58	0.06
SM (d42:2), 811.4→184.2	26.18	16.55	3.78	0.10
SM (d42:2), 811.5→184.2	17.54	10.93	2.43	0.08
Group				
Saturated	9.48	40.21	12.19	0.10
Unsaturated	64.99	90.26	7.82	0.05
Monounsaturated	12.59	39.20	6.51	0.02
Polyunsaturated	57.05	61.83	6.65	0.61

^1^Intensity ion counts multiplied by 1000. ^2^CON, control, no rumen protected Met, *n* = 4. ^3^MET, rumen protected Met, *n* = 5. ^4^SEM multiplied by 1000. *Variables for Methods 3 and 5 were not statistically different.

### Endometrial biopsy lipid analysis at 73 DIM

One PC was extracted for method 1 accounting for 89.7% of the total variability. Three PCs were extracted for methods 2 and 3 accounting for 83.4 and 82.6%, respectively, of the total variability. Finally, two PCs were extracted for methods 4 and 5 accounting for 82.0 and 83.9%, respectively, of the total variability (Table 8). Principal component one for method 1 at 73 DIM represents animals whose uterine biopsies contain mainly DG lipids. While group identification could not be accomplished for PC1 of method 2, PC2 represents animals that have higher abundances of TG lipid amounts. Moreover, PC3 represents animals that have higher DG lipid amounts. Finally, no groups were discovered for PC1 of method 5 at 73 DIM, however, PC2 represents animals that have higher DG lipid amounts. Analysis of lipids of interest according to the PCA for 73 DIM are in Table 9. Relative amounts of saturated lipids were higher (*p* = 0.06) for cows in MET compared to cows in CON. Likewise, cows in MET tended (*p* = 0.10) to have increased amounts of unsaturated lipids, specifically DG lipids, than cows in CON. Cows in MET had increased (*p* ≤ 0.05) monounsaturated lipid amounts compared to cows in CON. Tentative attributions for lipids of interest for endometrial biopsies obtained at 73 DIM based on PCA are in Supplementary Table S7. The extracted loadings (Method 1: PC1 loading ≥0.078854; Method 2: PC1 loadings ≥0.10085, PC2 loadings ≥0.1695, and PC3 loadings ≥0.21204; Method 3: PC1 loadings ≥0.099032, PC2 loadings ≥0.14595, and PC3 loadings ≥0.16354; Method 4: PC1 loadings ≥0.090057 and PC2 loadings ≥0.13231; Method 5: PC1 loadings ≥0.14999 and PC2 loadings ≥0.30029) are in Supplementary Table S8. Scores plots for methods 1–5 for lipid markers in uterine biopsies retrieved at 73 DIM are found in the Supplementary Figures S2–S6.

**Table 8:** Principal components (PC) of each method, proportion (p), and cumulative proportion (Cp) of the principal component analysis for uterine biopsies retrieved at 73 DIM.

Method	p	Cp
Method 1		
PC1*	89.7	89.7
PC2	5.4	95.0
PC3	2.4	97.4
PC4	1.2	98.6
Method 2		
PC1*	55.5	55.5
PC2*	16.6	72.1
PC3*	11.3	83.4
PC4	7.1	90.5
Method 3		
PC1*	55.9	55.9
PC2*	14.7	70.6
PC3*	11.9	82.6
PC4	7.1	89.6
Method 4		
PC1*	65.2	65.2
PC2*	16.8	82.0
PC3	6.4	88.5
PC4	5.4	93.9
Method 5		
PC1*	67.6	67.6
PC2*	16.3	83.9
PC3	4.3	88.2
PC4	2.7	90.8

*Extracted principal components.

**Table 9:** Least squares means and associated standard errors of normalized lipid marker intensity ion counts^1^ for uterine endometrial biopsies obtained at 73 DIM from Holstein cows supplemented with rumen-protected Met (MET) or not (CON) 21 days before calving until 73 DIM.

	Treatment			*p* value
Variable	CON^2^	MET^3^	SEM^4^	
Method 2, *m/z*				
DG (30:3); PC (18:1); PE (22:1); LysoPE (22:1), 535.9→206.6	2.75	4.82	0.61	0.02
DG (30:3); PC (18:1); PE (22:1); LysoPE (22:1), 536→206.7	4.51	10.72	1.93	0.03
PC (P-20:0); PE (22:1); LysoPE (22:1), 536.1→206.8	7.00	19.76	3.75	0.02
DG (30:2); PG (20:2); PA (24:2), 537→207.7	2.26	3.61	0.47	0.04
DG (36:8), 609.8→280.5	2.24	3.56	0.45	0.04
DG (36:7); PG (24:0), 611→282.2	4.42	10.49	2.75	0.11
DG (36:7); PG (24:0), 611.9→282.6	2.23	3.12	0.45	0.15
DG (36:7); PG (24:0), 612→283.2	2.95	4.76	0.87	0.13
DG (36:7); PG (24:0), 612.1→283.2	3.66	7.23	1.65	0.12
Method 3, *m/z*				
PC (34:2), 758.2→184.2	23.77	19.96	1.80	0.12
Method 4, *m/z*				
non-attributed, 537→282.2	18.23	57.55	11.49	0.02
non-attributed, 537.1→282.2	19.73	63.16	12.15	0.02
non-attributed, 537.2→282.2	17.44	55.78	10.82	0.02
PC (38:5); PI-CER (d36:1), 808.7→184.2	1.33	1.18	0.08	0.14
SM (d42:3), 811.7→184.2	2.77	2.16	0.21	0.05
PC (38:3), 812.8→184.2	2.07	1.78	0.11	0.06
PC (38:1), 816.5→184.2	8.79	7.40	0.71	0.15
Group				
Saturated	20.25	45.36	9.37	0.06
Unsaturated	70.74	100.50	12.95	0.10
Monounsaturated	24.38	43.88	5.89	0.02
Polyunsaturated	50.54	62.89	7.02	0.19

^1^Intensity ion counts multiplied by 1000. ^2^CON, control, no rumen protected Met, *n* = 5. ^3^MET, rumen protected Met, *n* = 8. ^4^SEM multiplied by 1000. *Variables for Methods 1 and 5 were not statistically different.

## Discussion

We aimed to assess the effects of supply of RPM during the transition period until around peak of lactation on lipid profiles of the preimplantation embryo and postpartum endometrial tissue of Holstein cows. We postulated that the lipid profiles, as well as unsaturation level, would be different in embryos and uterine tissue from cows fed RPM when compared with cows in CON. Our hypothesis was corroborated through the demonstration of increased PC and TG content, and polyunsaturated fatty acids in embryos from cows fed RPM in comparison with embryos from cows in CON. Additionally, feeding RPM also increased unsaturated and monounsaturated fatty acids in the endometrium at 30 DIM and at 73 DIM.

Phosphatidylcholine and sphingolipids are common lipids present in mammalian membranes, while TG are commonly stored in the cell cytoplasm (40). Unfortunately, the extent of research regarding the specific lipid classes present in the bovine embryo is limited. In the current study, embryos from cows supplemented with RPM showed an increase in TG and PC lipid content with an overall increase in polyunsaturated lipids. Ferreira et al. (41) reported similar results regarding an increase in PC lipids that contained a high degree of unsaturation for bovine embryos. Likewise, an evaluation of the fatty acid composition of lipids in cattle, sheep, and pig oocytes revealed that TG lipids contained the greatest fatty acid-rich fraction by mass (42). A possible explanation for the increased TG concentrations in the embryos may be a result of the RPM supplementation enabling cows to efficiently export TG from the liver, possibly increasing fatty acid concentration in blood (22). Embryos, like any other cell type, can generate lipid droplets in response to increase fatty acid availability (43). Increased embryonic lipid content, although detrimental to cryopreservation, indicates that these embryos have surplus energy substrates that could be utilized for other physiological processes (44). A recent study observed higher cleavage and blastocyst rates in embryos from large follicles that also had increased cytoplasmic lipid content as compared to small follicles, further denoting the importance of lipids during embryonic development (45). Additionally, lipid profiles in *Bos indicus* and *Bos taurus* suggest contributions toward embryonic development and changes in pre-ovulation (44).

Previous studies have demonstrated the importance of lipids in the uterine environment as well as the influence on embryo implantation to improve fertility (46, 47). Lysophosphatidic acid (LPA), a phospholipid derivative, may be an important factor in embryo implantation: LPA deficient mice had smaller embryos with decreased implantation and lower prostaglandin components in the uterus, all factors that contribute to reduced embryo vitality (47). The metabolism of Met results in S-adenosylmethionine, which is a major methylation agent, leading to methylation of phospholipids into phosphatidylcholine, which may explain the increase in PC lipids found in embryos from RPM supplemented cows (48, 49).

Ruminant reproduction requires a substantial amount of energy, as stressed by Mattos et al. (50), with the most important sources of energy being derived from the fatty acids in lipids. The energy usage by the ruminant reproductive tract occurs mainly through providing precursors for steroids, ultimately impacting pregnancy rates (50). In the endometrium, the platelet-activation factor rapidly hydrolyzes PC, resulting in the formation of DG (51), which serves as signal messengers and activate cellular events during implantation (52). For the current study, there was decreased PC lipids at 15 DIM with a notable increase in DG lipids in the endometrial tissue at 30 and 73 DIM with few to no PC lipids that were detected. It is possible that the PC lipids were hydrolyzed more efficiently in the cows supplemented with RPM, which could lead to increased signaling and improved implantation. Additionally, DG can further be metabolized by diacylglycerol/monoacylglycerol lipase, resulting in the formation of non-esterified fatty acids (53), such as arachidonic acid, leading to prostaglandin production. Weems et al. (54) described how prostaglandin E2 stimulated the secretion of progesterone in the bovine corpus luteum *in vitro* by day 200, showing that the luteotropic support of the hormone. Protein kinase C has been observed in gonadal tissue and can aid in secretory functions (55). Diacylglycerol increases protein kinase C enzymatic sensitivity 16-fold in pig ovarian cytosol, possibly resulting in increased endocrine secretions (55). Likewise, as previously stated, LPA is an important component for embryo implantation in mice. Ye et al. (56) modeled the importance of diacylglycerol for the formation of LPA which can also aid in uterine receptivity, oocyte maturation, and angiogenesis of the placenta and uterus. Based on these studies, DG can be considered an important component for all aspects of reproduction. Additionally, feeding dairy cattle with monounsaturated fatty acids has been reported to result in increased number of oocytes collected with a greater recovery rate (57).

Supplementation of RPM to multiparous Holstein cows resulted in increased ability to maintain pregnancy and overall embryonic size, leading to reduced embryo mortality (58). Thatcher et al. (59) hypothesized that the endometrial lipid status may act synergistically with bovine IFNτ, which is actively being produced by the conceptus, aiding in corpus luteum maintenance and embryonic survival through antagonizing the synthesis of prostaglandin F2α during the early stages of pregnancy. The typical voluntary waiting period for dairy cows to be bred is between 50 and 60 days after calving (60). The observed increase in lipids around this time further confirms the importance of lipids in the bovine uterus and embryos to maintain a viable pregnancy. Additionally, the increase in monounsaturated fatty acids in the endometrium at 30 DIM and at 73 DIM as a result of supplementation with RPM further corroborates the theory of SCD-1 being the link between lipid metabolism and sulfur amino acids. Furthermore, this is the first time this link is demonstrated to happen in the bovine endometrium. The enzyme SCD-1 is involved in the synthesis of monounsaturated fatty acids, and it was previously reported to increase the availability of sulfur amino acids (33). Thus, it is likely that the increase in monounsaturated fatty acids in the endometrial tissue is a response to increase SCD-1 due to the increased availability of Met.

The current study provides a comprehensive analysis of lipid profiles found in uterine endometrial tissue and preimplantation embryos from cows supplemented with RPM. Lipid profiling of the preimplantation embryos revealed an increase of TG, mainly polyunsaturated, which could be utilized for energy by the developing embryo. Moreover, the notable increase in DG and monounsaturated lipids signifies the possibility of the uterine endometrium in cows supplemented with RPM to assist with signaling, embryo implantation, and embryo growth. Therefore, providing RPM to Holstein cows during the transition period and early lactation may alter the lipid profiles of preimplantation embryos and the uterine endometrium, possibly leading to improved embryonic development and survival.

## Data availability statement

The raw data supporting the conclusions of this article will be made available by the authors, without undue reservation.

## Ethics statement

The animal study was approved by University of Illinois (Urbana-Champaign) Institutional Animal Care and Use Committee (no. 13023). The study was conducted in accordance with the local legislation and institutional requirements.

## Author contributions

SS: Data curation, Investigation, Project administration, Writing – original draft. AG: Validation, Visualization, Writing – review & editing. DV-A: Data curation, Formal analysis, Investigation, Writing – review & editing. CF: Formal analysis, Investigation, Writing – review & editing. MR: Investigation, Writing – review & editing. MW: Resources, Validation, Writing – review & editing. DL: Resources, Visualization, Writing – review & editing. FC: Conceptualization, Funding acquisition, Project administration, Resources, Supervision, Writing – review & editing.

## Footnotes

^1^http://www.maldi-msi.org

^2^www.metaboanalyst.ca

^3^http://www.lipidmaps.org

^4^https://metlin.scripps.edu/index.php
