# Supplementary material for: Rumen-protected methionine supplementation alters lipid profile of preimplantation embryo and endometrial tissue of Holstein cows
Source: Front Vet Sci. 2024 Jan 17;10:1301986. doi: 10.3389/fvets.2023.1301986 (PMC10827937; doi:10.3389/fvets.2023.1301986)
Supplement: Supplementary file 1 [file Data_Sheet_1.docx]

**APPENDIX A: SUPPLEMENTARY MATERIAL**

| **Table S1**. Tentative attributions for embryo lipid markers retrieved at 73 DIM. | |
| --- | --- |
| Detected *m/z* | Tentative Attribution^1, 2^ |
| 708.90 | [PC(P-30:2)+Na]^+^* |
| 710.90 | [PC(P-30:1)+Na]^+^ |
| 714.80 | [PC(O-30:0)+Na]^+^ |
| 715.80 | [TG(42:4)+H]^+^; [TG(40:1)+Na]^+^ |
| 725.51 | [SM(d34:1)+Na]^+^** |
| 732.90 | [PC(32:1)+H]^+^ |
| 782.50 | [PC(36:4)+H]^+^; [PC(34:1)+Na]^+^ |
| 787.50 | [SM(d40:1) +H]^+^**; [TG(44:1)+K]^+^ |
| 827.50 | [TG(50:4)+H]^+^; [TG(48:1)+Na]^+^ |
| 872.90 | [PC(42:1)+H]^+^; [PC(P-42:4)+Na]^+^ |
| 874.90 | [PC(42:0)+H]^+^; [PC(O-42:4)+Na]^+^ |
| 875.90 | [TG(54:8)+H]^+^; [TG(52:5)+Na]^+^ |
| 879.50 | [TG(54:6)+H]^+^; [TG(52:3)+Na]^+^ |
| 889.80 | [TG(54:1)+H]^+^; [TG(54:12)+Na]^+^; [TG(52:6)+K]^+^ |
| 890.00 | [TG(54:1)+H]^+^; [TG(52:6)+K]^+^ |
| 891.80 | [TG(54:0)+H]^+^; [TG(54:11)+Na]^+^; [TG(52:5)+K]^+^ |
| 896.60 | [PC(44:3)+H]^+^; [PC(42:0)+Na]^+^ |
| 906.71 | non-attributed^3^ |
| 907.60 | [TG(56:6)+H]^+^; [TG(54:3)+Na]^+^; [TG(54:11)+K]^+^ |
| 1036 | [TG(66:12)+H]^+^; [TG(64:9)+Na]^+^; [TG(64:17)+K]^+^; [TG(62:3)+K]^+^ |
| * Tentative attributions based on the detection of m/z 710.9 due to lipid not being listed in the LipidMaps database.  ** Sphingenine base.  ^1^All lipids were tentatively attributed based on the LipidMaps database considering up to 0.3 Da of difference from the theoretical mass except m/z 708.9 and m/z 732.9, which were up to 0.4 Da of difference from the theoretical mass.  ^2^Abbreviations: PC = phosphatidylcholine; SM = sphingomyelin; TG = triacylglycerol; ‘O-‘ prefix = presence of alkyl ether substituent; ‘P-‘ prefix = presence of 1Z-alkenyl ether (Plasmalogen) substituent.  ^3^Detected mass does not have attributed lipid according to LipidMaps Database. | |

| **Table S2.** Loadings for extracted PCs from embryos retrieved at 73 DIM. | | | | |
| --- | --- | --- | --- | --- |
| Lipid Attribution, *m/z* | Loadings | | | |
|  | PC1 | PC2 | PC3 | PC4 |
| [PC(P-30:2)+Na]^+^, 708.90 | -0.02553 | 0.2094* | 0.067397 | -0.08207 |
| [PC(P-30:1)+Na]^+^, 710.90 | 0.015795 | 0.099157 | 0.19717* | -0.01483 |
| [PC(O-30:0)+Na]^+^, 714.80 | 0.03144 | 0.08519 | 0.21261* | -0.0358 |
| [TG(42:4)+H]^+^; [TG(40:1)+Na]^+^, 715.80 | -0.00959 | 0.050163 | 0.2305* | 0.03359 |
| [SM(d34:1)+Na]^+^, 725.51 | -0.04028 | 0.112 | 0.012998 | -0.24116* |
| [PC(32:1)+H]^+^, 732.90 | -0.01979 | -0.06936 | 0.070696 | 0.26425* |
| [PC(36:4)+H]^+^; [PC(34:1)+Na]^+^, 782.50 | 0.00659 | 0.048021 | -0.01326 | -0.28234* |
| [SM(d40:1) +H]^+^; [TG(44:1)+K]^+^, 787.50 | 0.037169 | 0.026102 | -0.02989 | -0.27096* |
| [TG(50:4)+H]^+^; [TG(48:1)+Na]^+^, 827.50 | 0.16208* | 0.0178 | 0.040546 | 0.026245 |
| [PC(42:1)+H]^+^; [PC(P-42:4)+Na]^+^, 872.90 | 0.062582 | -0.20502* | -0.04687 | 0.014932 |
| [PC(42:0)+H]^+^; [PC(O-42:4)+Na]^+^, 874.90 | 0.050318 | -0.21739* | -0.01145 | -0.01364 |
| [TG(54:8)+H]^+^; [TG(52:5)+Na]^+^, 875.90 | 0.043132 | -0.21933* | -0.02285 | -0.01805 |
| [TG(54:6)+H]^+^; [TG(52:3)+Na]^+^, 879.50 | 0.1597* | 0.020988 | -0.04363 | 0.058251 |
| [TG(54:1)+H]^+^; [TG(54:12)+Na]^+^; [TG(52:6)+K]^+^, 889.80 | 0.021502 | 0.10304 | 0.21591* | 0.063981 |
| [TG(54:1)+H]^+^; [TG(52:6)+K]^+^, 890.00 | -0.04114 | -0.06088 | -0.01762 | 0.23844* |
| [TG(54:0)+H]^+^; [TG(54:11)+Na]^+^; [TG(52:5)+K]^+^, 891.80 | 0.089997 | -0.0634 | 0.19442* | -0.02529 |
| [PC(44:3)+H]^+^; [PC(42:0)+Na]^+^, 896.60 | 0.1601* | 0.02304 | -0.02526 | 0.013761 |
| non-attributed, 906.71 | 0.15459* | 0.002509 | -0.0815 | 0.065127 |
| [TG(56:6)+H]^+^; [TG(54:3)+Na]^+^; [TG(54:11)+K]^+^, 907.60 | 0.15469* | 0.061866 | -0.04206 | 0.046579 |
| [TG(66:12)+H]^+^; [TG(64:9)+Na]^+^; [TG(64:17)+K]^+^; [TG(62:3)+K]^+^, 1036 | 0.043428 | -0.20878* | -0.02373 | -0.02275 |
| * Loadings used for interpretation of PCA. | | | | |

| **Table S3.** Tentative attributions for relevant MRMs (parent -> fragment) for uterine biopsy lipid markers retrieved at 15 DIM. | |
| --- | --- |
| Method | Tentative Attribution^1^ |
| **Method 1**, *m/z* |  |
| 480.7 -> 223.4 | PA (20:0) |
| 481.7 -> 224.4 | PC (O-16:0); PE (18:0); LysoPE (18:0); PS (P-16:0/0:0) |
| 554.8 -> 281.5 | LysoPE (24:6); PS (20:0); CER (d34:1) |
| 556.9 -> 283.6 | CER (d18:0/16:0(2OH)) |
| 582.1 -> 206.8 | PS (22:0); CER (36:1) |
| 628.6 -> 371.3 | PE-CER (d32:3) |
| 631.9 -> 358.6 | PA (P-18:0/14:1) |
| 685.9 -> 428.6 | PA (O-36:3); PA (P-36:2); PE-CER (d36:3) |
| 701.6 -> 428.3 | DG (44:4); PA (36:2) |
| **Method2**, *m/z* |  |
| 432.1 -> 134.8 | non-attributed |
| 432.2 -> 106.9 | non-attributed |
| 432.2 -> 110.9 | non-attributed |
| 554 -> 208.7 | LysoPE (24:6); PS (20:0); CER (d34:1) |
| 555 -> 209.7 | PG (20:0); CER (d34:1) |
| 612.7 -> 283.4 | PI (P-20:0) |
| 614 -> 272.7 | non-attributed |
| 627.8 -> 282.5 | non-attributed |
| 628.9 -> 283.6 | PE-CER (d32:3) |
| 684.7 -> 355.4 | PE (32:4) |
| 685.6 -> 356.3 | PA (O-36:3); PA (P-36:2); PE-CER (d36:3) |
| 757.6 -> 428.3 | PG (O-36:4); PG (P-36:3); PA (40:2) |
| 850 -> 550.7 | TG (52:7); PG (42:7); PG (O-42:0); PI (O-36:2); PI (P-36:1) |
| 875.9 -> 576.6 | TG (54:7); PG (44:7); PI (P-38:1) |
| 878 -> 604.7 | TG (54:7); PG (44:7); PI (O-38:2); PI (P-38:1) |
| **Method 3**, *m/z* |  |
| 524.6-> 184.2 | PC (18:0) |
| 630.8 -> 281.5 | DG (36:0) |
| 701.7 -> 184.2 | SM (d34:2) |
| 703.6 -> 354.3 | SM (d34:1); PC (30:0); DG (42:3); PA (36:1) |
| 704.7 -> 355.4 | PC (30:1) |
| 705.7 -> 356.4 | non-attributed |
| 732.4 -> 184.2 | SM (d36:0); PC (32:1) |
| 735.6 -> 184.2 | PC (32:0) |
| 746.4 -> 184.2 | PC (O-34:1); PC (P-34:0) |
| 759.7 -> 184.2 | SM (d38:1) |
| 760.7 -> 184.2 | PC (34:1) |
| 761.6 -> 184.2 | SM (d38:0) |
| 770.7 -> 184.2 | PC (O-36:3); PC (P-36:2) |
| 775.4 -> 184.2 | PC (P-36:1) |
| 777.6 -> 428.3 | PA (44:6); PC (36:7); PE (P-40:6) |
| 777.9 -> 432.6 | TG (44:1); PG (36:1); PI (30:3); PA (42:6) |
| **Method 4**, *m/z* |  |
| 410 -> 132.7 | non-attributed |
| 454.2 -> 70.9 | PC (12:0); PC (O-14:0); PE (16:0); LysoPE (16:0) |
| 480.9 -> 282.2 | PA (20:0) |
| 481 -> 282.2 | CER (d30:1) |
| 520.2 -> 282.2 | non-attributed |
| 554.4 -> 266.4 | CER (d34:1) |
| 554.9 -> 282.2 | CER (d34:1) |
| 555.4 -> 282.2 | non-attributed |
| 586.2 -> 88.9 | non-attributed |
| 613.2 -> 282.2 | non-attributed |
| 759.5 -> 282.2 | SM (d38:1) |
| 777.3 -> 282.2 | PA (44:6) |
| 789.7 -> 184.2 | PC (36:0); PC (P-38:6) |
| 808.8 -> 184.2 | PC (38:5); PI-CER (d36:1) |
| 809.7 -> 184.2 | PC (38:5); PI-CER (d36:1) |
| 810.8 -> 184.2 | PC (38:4); PI-CER (d36:0) |
| **Method 5**, *m/z* |  |
| 538 -> 283.2 | PS (O-20:0) |
| 539.5 -> 283.2 | DG (30:0); PG (20:0) |
| 540.1 -> 283.2 | PC (20:5) |
| 612.3 -> 283.2 | oxidized glutathione |
| Abbreviations: CER = Ceramide; DG = Diacylglycerol; PA = Phosphatidic acid; PC = Phosphatidylcholine; PE = Phosphatidyletanolamine; PG = Phosphatidylglycerol; PI = Phosphatidylinositol; PS = Phosphatidylserine; SM = Sphingomyelin; TG = Triacylglycerol; ‘O-‘ prefix = presence of alkyl ether substituent; ‘P-‘ prefix = presence of 1Z-alkenyl ether (Plasmalogen) substituent  ^1^Only protonated (positive ion mode) and deprotonated (negative ion mode) molecules were considered. LipidMaps database was interrogated for lipids with structure compatible with the scan mode used considering up 0.5 Da of difference. If no match was found, other lipids listed at LipidMaps and metabolites listed at Metlin, considering up to 1.0 Da of difference, were included as tentative attributions. Mixtures of isomeric lipids may occur at the same MRM. Only one MRM has been listed for MRMs of similar m/z both for parent and fragment; We consider them to be the same lipid(s) due to the low resolution power of the mass spectrometer used. | |

| **Table S4.** Loadings for extracted PCs from uterine biopsies retrieved at 15 DIM. | | |
| --- | --- | --- |
| Variable | Loadings | |
|  | PC1 | PC2 |
| **Method 1,** *m/z* |  |  |
| PA (20:0), 480.7 -> 223.4 | 0.077447* |  |
| PC (O-16:0); PE (18:0); LysoPE (18:0);PS (P-16:0/0:0), 481.7 -> 224.4 | 0.077435* |  |
| LysoPE (24:6); PS (20:0); CER (d34:1), 554.8 -> 281.5 | 0.077437* |  |
| CER (d18:0/16:0(2OH)), 556.9 -> 283.6 | 0.07745* |  |
| PS (22:0); CER (36:1), 582.1 -> 206.8 | 0.077473* |  |
| PE-CER (d32:3), 628.6 -> 371.3 | 0.077458* |  |
| PA (P-18:0/14:1), 631.9 -> 358.6 | 0.077464* |  |
| PA (O-36:3); PA (P-36:2); PE-CER (d36:3), 685.9 -> 428.6 | 0.077463* |  |
| DG (44:4); PA (36:2), 701.6 -> 428.3 | 0.077452* |  |
| **Method 2,** *m/z* |  |  |
| non-attributed, 432.1 -> 134.8 | -0.0032513 | 0.17827* |
| non-attributed, 432.2 -> 106.9 | -0.0038316 | 0.1781* |
| non-attributed, 432.2 -> 110.9 | -0.01201 | 0.17326* |
| non-attributed, 432.2 -> 134.9 | -0.0030881 | 0.17975* |
| non-attributed, 432.3 -> 107.0 | -0.013443 | 0.17435* |
| LysoPE (24:6); PS (20:0); CER (d34:1), 554 -> 208.7 | -0.094111* | -0.0089101 |
| PG (20:0); CER (d34:1), 555 -> 209.7 | -0.094075* | -0.010197 |
| PI (P-20:0), 612.7 -> 283.4 | -0.093958* | -0.0071977 |
| non-attributed, 614 -> 272.7 | -0.093893* | -0.0094803 |
| non-attributed, 627.8 -> 282.5 | -0.093997* | -0.010075 |
| PE-CER (d32:3), 628.9 -> 283.6 | -0.094049* | -0.011156 |
| PE (32:4), 684.7 -> 355.4 | -0.094032* | -0.011176 |
| PA (O-36:3); PA (P-36:2); PE-CER (d36:3), 685.6 -> 356.3 | -0.093927* | -0.010199 |
| PG (O-36:4); PG (P-36:3); PA (40:2), 757.6 -> 428.3 | -0.093961* | -0.0095323 |
| TG (52:7); PG (42:7); PG (O-42:0); PI (O-36:2); PI (P-36:1), 850 -> 550.7 | 0.003314 | -0.17921* |
| TG (54:7); PG (44:7); PI (P-38:1), 875.9 -> 576.6 | 0.0034138 | -0.17397* |
| TG (54:7); PG (44:7); PI (O-38:2); PI (P-38:1), 878 -> 604.7 | 0.017534 | -0.17815* |
| **Method 3,** *m/z* |  |  |
| PC (18:0), 524.6 -> 184.2 | 0.095679* | -0.0014537 |
| DG (36:0), 630.8 -> 281.5 | 0.095667* | -0.0016222 |
| SM (d34:2), 701.7 -> 184.2 | 0.095682* | -0.0048856 |
| SM (d34:1); PC (30:0); DG (42:3); PA (36:1), 703.6 -> 354.3 | 0.095562* | -0.00092218 |
| PC (30:1), 704.7 -> 355.4 | 0.095599* | -0.0013151 |
| non-attributed , 705.7 -> 356.4 | 0.095596* | -0.0034002 |
| SM (d36:0); PC (32:1), 732.4 -> 184.2 | 0.011759 | 0.14156* |
| PC (32:0), 735.6 -> 184.2 | 0.023448 | -0.14761* |
| PC (O-34:1); PC (P-34:0), 746.4 -> 184.2 | -0.02512 | 0.14448* |
| PC (O-34:1); PC (P-34:0), 746.5 -> 184.2 | -0.010005 | 0.14829* |
| SM (d38:1), 759.7 -> 184.2 | 0.0079709 | 0.14761* |
| PC (34:1), 760.7 -> 184.2 | -0.016557 | 0.15191* |
| PC (34:1), 760.8 -> 184.2 | 0.02745 | 0.14402* |
| SM (d38:0), 761.6 -> 184.2 | -0.032017 | 0.14365* |
| PC (O-36:3); PC (P-36:2), 770.7 -> 184.2 | 0.095683* | 0.00056504 |
| PC (P-36:1), 775.4 -> 184.2 | 0.023861 | 0.14256* |
| PA (44:6); PC (36:7); PE (P-40:6), 777.6 -> 428.3 | 0.095572* | -0.00081625 |
| TG (44:1); PG (36:1); PI (30:3); PA (42:6), 777.9 -> 432.6 | 0.095559* | -0.00067994 |
| **Method 4,** *m/z* |  |  |
| non-attributed, 410 -> 132.7 | 0.087916* | 0.0042966 |
| PC (12:0); PC (O-14:0); PE (16:0); LysoPE (16:0), 454.2 -> 70.9 | 0.087758* | 0.00042023 |
| PA (20:0), 480.9 -> 282.2 | 0.039353 | -0.16734* |
| CER (d30:1), 481 -> 282.2 | 0.041939 | -0.1656* |
| CER (d30:1), 481.1 -> 282.2 | 0.032323 | -0.17478* |
| CER (d30:1), 481.2 -> 282.2 | 0.038947 | -0.1683* |
| non-attributed, 520.2 -> 282.2 | 0.087674* | 0.0092541 |
| CER (d34:1), 554.4 -> 266.4 | 0.087692* | 0.007086 |
| CER (d34:1), 554.9 -> 282.2 | 0.050191 | -0.15323* |
| non-attributed, 555.4 -> 282.2 | 0.087655* | -0.0054699 |
| non-attributed, 586.2 -> 88.9 | 0.087572* | 0.012301 |
| non-attributed, 613.2 -> 282.2 | 0.087608* | -0.0012227 |
| SM (d38:1), 759.5 -> 282.2 | 0.087559* | 0.00823 |
| PA (44:6), 777.3 -> 282.2 | 0.087589* | 0.0030725 |
| PC (36:0); PC (P-38:6), 789.7 -> 184.2 | 0.019234 | 0.15073* |
| PC (38:5); PI-CER (d36:1), 808.8 -> 184.2 | 0.043995 | -0.15222* |
| PC (38:5); PI-CER (d36:1), 809.7 -> 184.2 | -0.045353 | -0.15243* |
| PC (38:4); PI-CER (d36:0), 810.8 -> 184.2 | -0.038387 | -0.15385* |
| **Method 5,** *m/z* |  |  |
| PS (O-20:0), 538 -> 283.2 | 0.14611* | 0.039607 |
| DG (30:0); PG (20:0), 539.3 -> 283.2 | 0.016622 | -0.27105* |
| DG (30:0); PG (20:0), 539.4 -> 283.2 | 0.014594 | -0.27063* |
| DG (30:0); PG (20:0), 539.5 -> 283.2 | 0.012635 | -0.27031* |
| PC (20:5), 540.1 -> 283.2 | 0.14703* | -0.028184 |
| oxidized glutathione, 612.3 -> 283.2 | 0.14552* | 0.047996 |
| * Loadings used for interpretation of PCA. | | |

| **Table S5.** Tentative attributions for relevant MRMs (parent -> fragment) for uterine biopsy lipid markers retrieved at 30 DIM. | |
| --- | --- |
| Method | Tentative Attribution^1^ |
| **Method 1**, *m/z* |  |
| 481 -> 207.7 | PA (20:0) |
| 481.9 -> 224.6 | PC (O-16:0); PE (18:0); LysoPE (18:0); PS (P-16:0/0:0) |
| 553.8 -> 280.5 | β-Cryptoxanthin |
| 554.1 -> 280.8 | LysoPE (24:6); PS (20:0); CER (d34:1) |
| 555.9 -> 282.6 | non-attributed |
| 582.1 -> 206.8 | PS (22:0); CER (36:1) |
| 611.7 -> 354.4 | DG (36:7) |
| 612 -> 354.7 | PI (P-20:0) |
| 627.9 -> 354.6 | non-attributed |
| 627.9 -> 370.6 | non-attributed |
| 629.8 -> 356.5 | PE-CER (d32:3) |
| 630 -> 356.7 | non-attributed |
| 686 -> 428.7 | PE (32:3); PA (O-36:3); PA (P-36:2); PE-CER (d36:3); GlcCER (d32:2) |
| 701.9 -> 428.6 | DG (44:4); PA (36:2) |
| 702.8 -> 429.5 | PC (30:2); PE (P-34:1); PS (30:3) |
| 703.8 -> 430.5 | DG (42:3); PA (36:1) |
| 787.8 -> 184.2 | SM (d40:1); PC (36:2) |
| **Method 2**, *m/z* |  |
| 432.2 -> 106.9 | non-attributed |
| 432.2 -> 110.9 | non-attributed |
| 555 -> 209.7 | PG (20:0); CER (d34:1) |
| 609.8-> 280.5 | DG (36:8) |
| 611.5 -> 270.2 | DG (36:7); PG (24:0) |
| 611.9 -> 282.6 | DG (36:7); PG (24:0) |
| 613 -> 283.7 | DG (36:6); PA (30:4) |
| 613.9 -> 272.6 | DG (36:6); PA (30:4) |
| 614 -> 272.7 | non-attributed |
| 628.9 -> 283.6 | non-attributed |
| 683.8 -> 354.5 | PI (20:0) |
| 684.2 -> 354.9 | PE (32:4) |
| 686.7 -> 345.4 | PE (32:3); PA (O-36:3); PA (P-36:2); PE-CER (d36:3); GlcCER (d32:2) |
| 700.8 -> 359.5 | PC (30:3); PE (O-34:3); PE (P-34:2); PS (30:4); GlcCER (d34:1) |
| 850 -> 550.7 | TG (52:7); PG (42:7); PI (O-36:2); PI (P-36:1) |
| 863.8 -> 590.5 | TG (52:0); PG (42:0); PI (36:2) |
| **Method 3**, *m/z* |  |
| 487.9-> 132.6 | Calyxinin and ceanin (sphingoid bases) |
| 705.7 -> 356.4 | PA (P-38:6) |
| 706.4 -> 184.2 | PC (O-38:4); PC (P-38:3) |
| 718.3 -> 184.2 | PC (O-32:1); PC (P-32:0) |
| 720.4 -> 184.2 | PC (O-32:1); PC (P-32:0) |
| 734 -> 184.2 | SM (d36:0) |
| 735.5 -> 184.2 | PC (32:0) |
| 759.5 -> 184.2 | SM (d38:1) |
| 763.8 -> 184.2 | PC (P-36:5) |
| 766.3 -> 184.2 | PC (O-36:5); PC (P-36:4) |
| 776.8 -> 431.5 | PC (36:7); PE (P-40:6) |
| 777.6 -> 428.3 | TG (44:1); PG (36:1); PI (30:3); PA (42:6) |
| 777.6 -> 432.3 | TG (44:1); PG (36:1); PI (30:3); PA (42:6) |
| 785.5 -> 184.2 | SM (d40:2) |
| 851.7 -> 502.4 | TG (52:6); PG (42:6) |
| **Method 4**, *m/z* |  |
| 352.7 -> 85.1 | 15(R),19(R)-hydroxy PGF2α; 9,11-methane-epoxy PGF1α; δ-12-PGD2; Dodecylphosphocholine |
| 388.7 -> 85.1 | 17-phenyl trinor PGF2α amide; 17-phenyl trinor PGF2α |
| 410.8 -> 85.1 | PA (16:0); LPA (16:0) |
| 447.1 -> 225.2 | 17-phenoxy trinor Prostaglandin F2α isopropyl ester |
| 475.8 -> 85.1 | PE (18:3); LysoPE (18:3) |
| 538.3 -> 282.2 | CER (d34:1) |
| 539.2 -> 282.2 | non-attributed |
| 540.2 -> 282.2 | CER (d34:0) |
| 583.4 -> 282.2 | non-attributed |
| 611.3 -> 282.2 | DG (36:7); PG (24:0) |
| 613.2 -> 282.2 | Non-attributed |
| 630.7 -> 445.5 | Non-attributed |
| 803.7 -> 184.2 | non-attributed |
| 811.4 -> 184.2 | SM (d42:2) |
| 816.8 -> 184.2 | PC (38:1) |
| **Method 5**, *m/z* |  |
| 407.8 -> 222.6 | non-attributed |
| 464.9 -> 283.2 | PA (20:0) |
| 538 -> 283.2 | PC (18:0); PC (O-20:0); PE (22:0); LysoPE (22:0); PS (P-20:0); CER (d34:1) |
| 666.5 -> 369.1 | GlcCER (d32:3) |
| 804.9 -> 241.3 | non-attributed |
| 807.4 -> 241.3 | PI (32:1) |
| Abbreviations: CER = Ceramide; DG = Diacylglycerol; PA = Phosphatidic acid; PC = Phosphatidylcholine; PE = Phosphatidyletanolamine; PG = Phosphatidylglycerol; PI = Phosphatidylinositol; PS = Phosphatidylserine; SM = Sphingomyelin; TG = Triacylglycerol; ‘O-‘ prefix = presence of alkyl ether substituent; ‘P-‘ prefix = presence of 1Z-alkenyl ether (Plasmalogen) substituent  ^1^Only protonated (positive ion mode) and deprotonated (negative ion mode) molecules were considered. LipidMaps database was interrogated for lipids with structure compatible with the scan mode used considering up 0.5 Da of difference. If no match was found, other lipids listed at LipidMaps and metabolites listed at Metlin, considering up to 1.0 Da of difference, were included as tentative attributions. Mixtures of isomeric lipids may occur at the same MRM. Only one MRM has been listed for MRMs of similar m/z both for parent and fragment; We consider them to be the same lipid(s) due to the low-resolution power of the mass spectrometer used. | |

| **Table S6.** Loadings for extracted PCs from uterine biopsies retrieved at 30 DIM. | | |
| --- | --- | --- |
| Variable | Loadings | |
|  | PC1 | PC2 |
| **Method 1,** *m/z* |  |  |
| PA (20:0), 481 -> 207.7 | 0.082897* | -0.0009931 |
| PC (O-16:0); PE (18:0); LysoPE (18:0); PS (P-16:0/0:0), 481.9 -> 224.6 | 0.082703* | -0.0098268 |
| β-Cryptoxanthin , 553.8 -> 280.5 | 0.082637* | 0.0099154 |
| LysoPE (24:6); PS (20:0); CER (d34:1), 554.1 -> 280.8 | 0.035122 | 0.16695* |
| non-attributed, 555.9 -> 282.6 | 0.082707* | -0.014755 |
| PS (22:0); CER (36:1), 582.1 -> 206.8 | 0.0829* | -0.010839 |
| DG (36:7), 611.7 -> 354.4 | 0.082782* | 0.012056 |
| PI (P-20:0), 612 -> 354.7 | 0.035631 | 0.16615* |
| non-attributed, 627.9 -> 354.6 | 0.036366 | 0.16645* |
| non-attributed, 627.9 -> 370.6 | 0.044434 | 0.15433* |
| non-attributed, 628 -> 354.7 | 0.022654 | 0.17736* |
| PE-CER (d32:3), 629.8 -> 356.5 | 0.082788* | 0.0074563 |
| non-attributed, 630 -> 356.7 | 0.041854 | 0.15901* |
| PE (32:3); PA (O-36:3); PA (P-36:2); PE-CER (d36:3); GlcCER (d32:2), 686 -> 428.7 | 0.035952 | 0.16239* |
| DG (44:4); PA (36:2), 701.9 -> 428.6 | 0.041176 | 0.15628* |
| PC (30:2); PE (P-34:1); PS (30:3), 702.8 -> 429.5 | 0.082916* | 0.01049 |
| DG (42:3); PA (36:1), 703.8 -> 430.5 | 0.082641* | -0.010796 |
| SM (d40:1); PC (36:2), 787.8 -> 184.2 | 0.028642 | -0.15724* |
| **Method 2,** *m/z* |  |  |
| non-attributed, 432.1 -> 106.8 | 0.00060549 | -0.14692* |
| non-attributed, 432.2 -> 106.9 | -0.0083653 | -0.14648* |
| non-attributed, 432.2 -> 110.9 | -0.0018655 | -0.14491* |
| non-attributed, 432.3 -> 107.0 | -0.0080815 | -0.14561* |
| PG (20:0); CER (d34:1), 555 -> 209.7 | 0.092405* | 0.0012313 |
| DG (36:8), 609.8 -> 280.5 | -0.013692 | 0.14623* |
| DG (36:7); PG (24:0), 611.5 -> 270.2 | 0.092401* | 0.0043867 |
| DG (36:7); PG (24:0), 611.9 -> 282.6 | 0.00011163 | 0.14761* |
| DG (36:6); PA (30:4), 613 -> 283.7 | 0.015945 | 0.14579* |
| DG (36:6); PA (30:4), 613.9 -> 272.6 | 0.092422* | 0.0014103 |
| non-attributed, 614 -> 272.7 | 0.092384* | -0.0022228 |
| non-attributed, 628.9 -> 283.6 | 0.092401* | -0.00091948 |
| PI (20:0), 683.8 -> 354.5 | -0.014515 | 0.14449* |
| PE (32:4), 684.2 -> 354.9 | -0.0036464 | 0.1452* |
| PE (32:3); PA (O-36:3); PA (P-36:2); PE-CER (d36:3); GlcCER (d32:2), 686.7 -> 345.4 | 0.092384* | -0.002556 |
| PC (30:3); PE (O-34:3); PE (P-34:2); PS (30:4); GlcCER (d34:1), 700.8 -> 359.5 | 0.092396* | 0.0037452 |
| TG (52:7); PG (42:7); PI (O-36:2); PI (P-36:1), 850 -> 550.7 | 0.092387* | 0.0051479 |
| TG (52:0); PG (42:0); PI (36:2), 863.8 -> 590.5 | 0.092404* | 0.003079 |
| **Method 3,** *m/z* |  |  |
| Calyxinin and ceanin (sphingoid bases), 487.9 -> 132.6 | 0.088451* | -0.0031426 |
| PA (P-38:6), 705.7 -> 356.4 | 0.08844* | -0.0018079 |
| PC (O-38:4); PC (P-38:3), 706.4 -> 184.2 | -0.0069848 | -0.15648* |
| PC (O-32:1); PC (P-32:0), 718.3 -> 184.2 | 0.01453 | 0.16665* |
| PC (O-32:1); PC (P-32:0), 720.4 -> 184.2 | 0.019719 | -0.1695* |
| SM (d36:0), 734 -> 184.2 | 0.01575 | -0.17068* |
| PC (32:0), 735.5 -> 184.2 | -0.0005473 | -0.17322* |
| SM (d38:1), 759.5 -> 184.2 | -0.034816 | -0.15775* |
| PC (P-36:5), 763.8 -> 184.2 | 0.088444* | 0.00061941 |
| PC (O-36:5); PC (P-36:4), 766.3 -> 184.2 | -0.0127 | 0.16252* |
| PC (O-36:5); PC (P-36:4), 766.5 -> 184.2 | 0.0015162 | 0.16928* |
| PC (36:7); PE (P-40:6), 776.8 -> 431.5 | 0.088451* | -0.002177 |
| TG (44:1); PG (36:1); PI (30:3); PA (42:6), 777.6 -> 428.3 | 0.088444* | -0.0018177 |
| TG (44:1); PG (36:1); PI (30:3); PA (42:6), 777.6 -> 432.3 | 0.088449* | -0.0019971 |
| TG (44:1); PG (36:1); PI (30:3); PA (42:6), 777.8 -> 432.5 | 0.08845* | -0.0021894 |
| TG (44:1); PG (36:1); PI (30:3); PA (42:6), 777.9 -> 432.6 | 0.088449* | -0.002119 |
| SM (d40:2), 785.5 -> 184.2 | -0.015131 | -0.17927* |
| TG (52:6); PG (42:6), 851.7 -> 502.4 | 0.08845* | -0.0021656 |
| **Method 4,** *m/z* |  |  |
| 15(R),19(R)-hydroxy PGF2α; 9,11-methane-epoxy PGF1α; δ-12-PGD2; Dodecylphosphocholine, 352.7 -> 85.1 | 0.0083536 | -0.12266* |
| 17-phenyl trinor PGF2α amide; 17-phenyl trinor PGF2α , 388.7 -> 85.1 | -0.0070143 | -0.12144* |
| PA (16:0); LPA (16:0), 410.8 -> 85.1 | -0.0091036 | -0.12231* |
| 17-phenoxy trinor Prostaglandin F2α isopropyl ester , 447.1 -> 225.2 | 0.0061826 | -0.12179* |
| PE (18:3); LysoPE (18:3), 475.8 -> 85.1 | 0.0082725 | -0.12239* |
| CER (d34:1), 538.3 -> 282.2 | 0.1006* | 0.012009 |
| CER (d34:1), 538.9 -> 282.2 | 0.10216* | 0.0087594 |
| CER (d34:1), 539 -> 282.2 | 0.098898* | 0.02466 |
| non-attributed, 539.2 -> 282.2 | 0.10336* | 0.0059062 |
| CER (d34:0), 540.2 -> 282.2 | 0.10106* | -0.019341 |
| non-attributed, 583.4 -> 282.2 | 0.01206 | -0.12273* |
| DG (36:7); PG (24:0), 611.3 -> 282.2 | 0.10203* | -0.019823 |
| Non-attributed, 613.2 -> 282.2 | 0.099578* | -0.032682 |
| Non-attributed, 630.7 -> 445.5 | -0.0053216 | -0.1219* |
| Non-attributed, 803.7 -> 184.2 | -0.01031 | -0.12371* |
| SM (d42:2), 811.4 -> 184.2 | -0.099954* | 0.002497 |
| SM (d42:2), 811.5 -> 184.2 | -0.099742* | -0.0059411 |
| PC (38:1), 816.8 -> 184.2 | -0.0080715 | -0.12363* |
| **Method 5,** *m/z* |  |  |
| non-attributed, 407.8 -> 222.6 | 0.14766* | 0.011728 |
| PA (20:0), 464.9 -> 283.2 | -0.051037 | 0.26192* |
| PC (18:0); PC (O-20:0); PE (22:0); LysoPE (22:0); PS (P-20:0); CER (d34:1), 538 -> 283.2 | 0.14766* | 0.006588 |
| GlcCER (d32:3), 666.5 -> 369.1 | 0.14776* | -1.49E-05 |
| non-attributed, 804.9 -> 241.3 | 0.012835 | -0.27869* |
| PI (32:1), 807.4 -> 241.3 | 0.063051 | 0.26296* |
| * Loadings used for interpretation of PCA. | | |

| **Table S7.** Tentative attributions for relevant MRMs (parent -> fragment) for uterine biopsy lipid markers retrieved at 73 DIM. | |
| --- | --- |
| Method | Tentative Attribution^1^ |
| **Method 1**, *m/z* |  |
| 535.8 -> 264.5 | DG (30:3) |
| 536.9 -> 265.6 | PC (18:1); PE (22:1); LysoPE (22:1) |
| 551.9 -> 280.6 | DG (P-32:1); PS (20:1) |
| 609.8 -> 338.5 | DG (36:8) |
| 611.6 -> 354.3 | DG (36:7) |
| 613.7 -> 356.4 | DG (36:6); PA (30:4) |
| 628.7 -> 371.4 | PE-CER (d32:3) |
| 701.5 -> 428.2 | DG (42:4); PA (36:2) |
| **Method 2**, *m/z* |  |
| 535.9 -> 206.6 | DG (30:3); PC (18:1); PE (22:1); LysoPE (22:1) |
| 536.1 -> 206.8 | PC (P-20:0); PE (22:1); LysoPE (22:1) |
| 537 -> 207.7 | DG (30:2); PG (20:2); PA (24:2) |
| 555.9 -> 210.6 | PG (20:0) |
| 609.8 -> 280.5 | DG (36:8) |
| 611 -> 282.2 | DG (36:7); PG (24:0) |
| 611.8 -> 270.5 | DG (36:7); PG (24:0) |
| 612.7 -> 271.4 | PI (P-20:0) |
| 612.7 -> 283.4 | PI (P-20:0) |
| 613.9 -> 272.6 | DG (36:6); PA (30:4) |
| 629.9 -> 284.6 | PE-CER (d32:3) |
| 685.9 -> 344.6 | PA (O-36:3); PA (P-36:2); PE-CER (d36:3) |
| 686.7 -> 345.4 | PE (32:3); PA (O-36:3); PA (P-36:2); PE-CER (d36:3); GlcCER (d32:2) |
| 848.1 -> 548.8 | TG (52:8); PS (P-42:6); PG (O-42:1) |
| 850 -> 550.7 | TG (52:7); PG (42:7); PG (O-42:0); PI (O-36:2); PI (P-36:1) |
| 850 -> 576.7 | TG (52:7); PG (42:7); PI (O-36:2); PI (P-36:1) |
| 875.7 -> 576.4 | TG (54:8); PG (44:8); PI (O-38:3); PI (P-38:2) |
| 876 -> 602.7 | TG (54:8); PG (44:8); PI (O-38:3); PI (P-38:2) |
| 876.9 -> 577.6 | PS (42:0) |
| 878 -> 578.7 | TG (54:7); PG (44:7); PI (O-38:2); PI (P-38:1) |
| 878 -> 604.7 | TG (54:7); PG (44:7); PI (O-38:2); PI (P-38:1) |
| 905.9 -> 606.6 | TG (56:7); PI (40:9); PI (O-40:2); PI (P-40:1) |
| **Method 3**, *m/z* |  |
| 410 -> 56.7 | PGF2α diethyl amide; Tafluprost (free acid); 1-Palmitoyl Lysophosphatidic Acid; N-arachidonoyl taurine |
| 438.3 -> 89 | PC (O-12:1); PE (P-16:0) |
| 444 -> 58.7 | non-attributed |
| 524.6 -> 184.2 | PC (18:0) |
| 701.7 -> 184.2 | SM (d34:2) |
| 703.5 -> 184.2 | SM (d34:1); PC (30:0) |
| 704.5 -> 184.2 | PC (30:1) |
| 720.8 -> 184.2 | PC (O-32:1); PC (P-32:0) |
| 732.8 -> 184.2 | SM (d36:0); PC (32:1) |
| 758.2 -> 184.2 | PC (34:2) |
| 759.5 -> 184.2 | SM (d38:1) |
| 761.6 -> 184.2 | SM (d38:0) |
| 784.6 -> 184.2 | PC (36:3) |
| 785.6 -> 184.2 | SM (d40:2) |
| 786.1 -> 184.2 | PC (36:2) |
| **Method 4**, *m/z* |  |
| 293.8 -> 97.1 | non-attributed |
| 454.2 -> 70.9 | PC (12:0); PC (O-14:0); PE (16:0); LysoPE (16:0) |
| 475.8 -> 85.1 | PE (18:3); LysoPE (18:3) |
| 520.2 -> 282.2 | non-attributed |
| 537.2 -> 282.2 | non-attributed |
| 548.4 -> 107.1 | PC (O-20:2); PC (20:2); PS (20:3) |
| 556.4 -> 282.2 | CER (d34:0); CER (t34:0) |
| 610.5 -> 266.4 | CER (d38:1) |
| 611.3 -> 282.2 | DG (36:7); PG (24:0) |
| 613.3 -> 282.2 | DG (36:6); PA (30:4) |
| 808.7 -> 184.2 | PC (38:5); PI-CER (d36:1) |
| 811.7 -> 184.2 | SM (d42:3) |
| 812.8 -> 184.2 | PC (38:3) |
| 814.9 -> 184.2 | PC (38:2); PI-CER (t34:0) |
| 815.5 -> 184.2 | SM (d42:1) |
| 816.5 -> 184.2 | PC (38:1) |
| **Method 5**, *m/z* |  |
| 537.7 -> 283.2 | DG (30:2); PG (20:2); PA (24:0) |
| 538.3 -> 283.2 | PC (18:0); PC (O-20:0); PE (22:0); LysoPE (22:0); PS (P-20:0); CER (d34:1) |
| 539.3 -> 283.2 | DG (30:1); PG (20:1) |
| 630.2 -> 283.2 | DG (36:0) |
| Abbreviations: CER = Ceramide; DG = Diacylglycerol; PA = Phosphatidic acid; PC = Phosphatidylcholine; PE = Phosphatidyletanolamine; PG = Phosphatidylglycerol; PI = Phosphatidylinositol; PS = Phosphatidylserine; SM = Sphingomyelin; TG = Triacylglycerol; ‘O-‘ prefix = presence of alkyl ether substituent; ‘P-‘ prefix = presence of 1Z-alkenyl ether (Plasmalogen) substituent  ^1^Only protonated (positive ion mode) and deprotonated (negative ion mode) molecules were considered. LipidMaps database was interrogated for lipids with structure compatible with the scan mode used considering up 0.5 Da of difference. If no match was found, other lipids listed at LipidMaps and metabolites listed at Metlin, considering up to 1.0 Da of difference, were included as tentative attributions. Mixtures of isomeric lipids may occur at the same MRM. Only one MRM has been listed for MRMs of similar m/z both for parent and fragment; We consider them to be the same lipid(s) due to the low-resolution power of the mass spectrometer used. | |

| **Table S8.** Loadings for extracted PCs from uterine biopsies retrieved at 73 DIM. | | | |
| --- | --- | --- | --- |
| Variable | Loadings | | |
|  | PC1 | PC2 | PC3 |
| **Method 1,** *m/z* |  | | |
| DG (30:3), 535.8 -> 264.5 | 0.079008* |  |  |
| PC (18:1); PE (22:1); LysoPE (22:1), 536.8 -> 265.5 | 0.078867* |  |  |
| PC (18:1); PE (22:1); LysoPE (22:1), 536.9 -> 265.6 | 0.07897* |  |  |
| DG (P-32:1); PS (20:1), 551.9 -> 280.6 | 0.079029* |  |  |
| DG (36:8), 609.8 -> 338.5 | 0.078917* |  |  |
| DG (36:7), 611.6 -> 354.3 | 0.078975* |  |  |
| DG (36:6); PA (30:4), 613.7 -> 356.4 | 0.079037* |  |  |
| PE-CER (d32:3), 628.7 -> 371.4 | 0.078854* |  |  |
| DG (42:4); PA (36:2), 701.5 -> 428.2 | 0.078868* |  |  |
| **Method 2,** *m/z* |  |  |  |
| DG (30:3); PC (18:1); PE (22:1); LysoPE (22:1), 535.9 -> 206.6 | -0.00073173 | -0.037724 | -0.21689* |
| PC (P-20:0); PE (22:1); LysoPE (22:1), 536 -> 206.7 | -0.0078914 | -0.032463 | -0.21843* |
| PC (P-20:0); PE (22:1); LysoPE (22:1), 536.1 -> 206.8 | -0.018699 | -0.032858 | -0.21379* |
| DG (30:2); PG (20:2); PA (24:2), 537 -> 207.7 | 0.0017452 | -0.017404 | -0.21904* |
| PG (20:0), 555.9 -> 210.6 | 0.10091* | -0.0019663 | -0.00081302 |
| DG (36:8), 609.8 -> 280.5 | 0.0062614 | -0.028247 | -0.21441* |
| DG (36:7); PG (24:0), 611 -> 282.2 | -0.017493 | -0.022301 | -0.21204* |
| DG (36:7); PG (24:0), 611.8 -> 270.5 | 0.10085* | -0.00073998 | 8.89E-05 |
| DG (36:7); PG (24:0), 611.9-> 282.6 | -0.0012196 | -0.0051756 | -0.21466* |
| DG (36:7); PG (24:0), 612 -> 283.2 | -0.013136 | -0.014976 | -0.21497* |
| DG (36:7); PG (24:0), 612.1 -> 283.2 | -0.0098319 | -0.025115 | -0.21443* |
| PI (P-20:0), 612.7 -> 271.4 | 0.10085* | -0.0033852 | 0.0011715 |
| PI (P-20:0), 612.7 -> 283.4 | 0.10086* | 0.0011438 | -0.0077951 |
| DG (36:6); PA (30:4), 613.9 -> 272.6 | 0.10085* | -0.0035307 | 0.00099438 |
| PE-CER (d32:3), 629.9 -> 284.6 | 0.10086* | -0.0030597 | 0.0018155 |
| PA (O-36:3); PA (P-36:2); PE-CER (d36:3), 685.5 -> 344.2 | 0.10086* | -0.0036149 | 0.0011988 |
| PA (O-36:3); PA (P-36:2); PE-CER (d36:3), 685.9 -> 344.6 | 0.10087* | -0.0036207 | 0.0014069 |
| PE (32:3); PA (O-36:3); PA (P-36:2); PE-CER (d36:3); GlcCER (d32:2), 686.7 -> 345.4 | 0.10088* | -0.0024669 | 0.001662 |
| TG (52:8); PS (P-42:6); PG (O-42:1), 848.1 -> 548.8 | -0.014584 | -0.17482* | 0.04843 |
| TG (52:7); PG (42:7); PG (O-42:0); PI (O-36:2); PI (P-36:1), 850 -> 550.7 | 0.021081 | -0.17147* | 0.055649 |
| TG (52:7); PG (42:7); PI (O-36:2); PI (P-36:1), 850 -> 576.7 | -0.0032157 | -0.17146* | 0.066973 |
| TG (54:8); PG (44:8); PI (O-38:3); PI (P-38:2), 875.7 -> 576.4 | -0.028648 | -0.1695* | 0.047555 |
| TG (54:8); PG (44:8); PI (O-38:3); PI (P-38:2), 876 -> 602.7 | -0.026825 | -0.17042* | 0.049595 |
| PS (42:0), 876.9 -> 577.6 | -0.025555 | -0.17089* | 0.049965 |
| TG (54:7); PG (44:7); PI (O-38:2); PI (P-38:1), 878 -> 578.7 | -0.0056087 | -0.17612* | 0.05326 |
| TG (54:7); PG (44:7); PI (O-38:2); PI (P-38:1), 878 -> 604.7 | -0.0030891 | -0.17645* | 0.055231 |
| TG (56:7); PI (40:9); PI (O-40:2); PI (P-40:1), 905.9 -> 606.6 | 0.006346 | -0.17556* | 0.051311 |
| **Method 3,** *m/z* |  |  |  |
| PGF2α diethyl amide; Tafluprost (free acid); 1-Palmitoyl Lysophosphatidic Acid; N-arachidonoyl taurine , 410 -> 56.7 | 0.099298* | 0.0037846 | 0.021807 |
| PC (O-12:1); PE (P-16:0), 438.3 -> 89 | 0.099267* | 0.0073764 | 0.018282 |
| non-attributed , 444 -> 58.7 | 0.099199* | 0.001393 | 0.010901 |
| PC (18:0), 524.6 -> 184.2 | 0.099307* | 0.0083504 | 0.016691 |
| SM (d34:2), 701.7 -> 184.2 | 0.099571* | 0.0025467 | 0.0090799 |
| SM (d34:1); PC (30:0), 703.4 -> 184.2 | 0.049977 | -0.065749 | -0.1663* |
| SM (d34:1); PC (30:0), 703.5 -> 184.2 | 0.040862 | -0.064301 | -0.17525* |
| PC (30:1), 703.6 -> 184.2 | 0.049119 | -0.071082 | -0.16354* |
| PC (30:1), 704.2 -> 184.2 | 0.051796 | -0.040138 | -0.16812* |
| PC (30:1), 704.3 -> 184.2 | 0.055261 | -0.050444 | -0.164* |
| PC (30:1), 704.4 -> 184.2 | 0.052472 | -0.053217 | -0.16976* |
| PC (30:1), 704.5 -> 184.2 | 0.042814 | -0.068976 | -0.17108* |
| PC (30:1), 704.8 -> 184.2 | 0.099476* | 0.013658 | 0.0019777 |
| PC (30:1), 705.8 -> 356.5 | 0.099032* | 0.007176 | 0.023821 |
| PC (O-32:1); PC (P-32:0), 720.8 -> 184.2 | 0.099131* | 0.0093951 | 0.02269 |
| SM (d36:0); PC (32:1), 732.8 -> 184.2 | 0.099363* | -0.0014104 | 0.022943 |
| PC (34:2), 758.2 -> 184.2 | -0.052211 | 0.14595* | -0.039223 |
| PC (34:2), 759 -> 184.2 | 0.051649 | 0.14773* | -0.010776 |
| SM (d38:1), 759.3 -> 184.2 | -0.023085 | 0.16918* | -0.032798 |
| SM (d38:1), 759.4 -> 184.2 | -0.031947 | 0.16334* | -0.057741 |
| SM (d38:1),759.5 -> 184.2 | 0.010104 | 0.16848* | -0.047386 |
| SM (d38:1), 759.7 -> 184.2 | 0.0443 | -0.063377 | 0.16389* |
| SM (d38:0), 761.6 -> 184.2 | -0.019074 | -0.093572 | 0.16823* |
| SM (d40:2), 784.6 -> 184.2 | -0.037025 | 0.15154* | -0.010411 |
| SM (d40:2), 785.6 -> 184.2 | 0.025631 | 0.15866* | -0.079867 |
| SM (d40:2), 785.9 -> 184.2 | -0.0079489 | 0.1546* | -0.045612 |
| PC (36:2), 786.1 -> 184.2 | -0.046916 | 0.14726* | -0.04031 |
| **Method 4,** *m/z* |  |  |  |
| non-attributed , 293.8 -> 97.1 | 0.090291* | -0.030321 |  |
| PC (12:0); PC (O-14:0); PE (16:0); LysoPE (16:0), 454.2 -> 70.9 | 0.090392* | -0.030747 |  |
| PE (18:3); LysoPE (18:3), 475.8 -> 85.1 | 0.090057* | -0.034958 |  |
| non-attributed, 520.2 -> 282.2 | 0.090593* | -0.02619 |  |
| non-attributed, 537 -> 282.2 | 0.060834 | 0.13412* |  |
| non-attributed, 537.1 -> 282.2 | 0.061291 | 0.13268* |  |
| non-attributed, 537.2 -> 282.2 | 0.061811 | 0.13231* |  |
| PC (O-20:2); PC (20:2); PS (20:3), 548.4 -> 107.1 | 0.090094* | -0.020433 |  |
| CER (d34:0); CER (t34:0), 556.4 -> 282.2 | 0.09037* | -0.031571 |  |
| CER (d38:1), 610.5 -> 266.4 | 0.090087* | -0.033096 |  |
| DG (36:7); PG (24:0), 611.3 -> 282.2 | 0.090302* | 0.013903 |  |
| DG (36:6); PA (30:4), 613.3 -> 282.2 | 0.09067* | -0.017939 |  |
| PC (38:5); PI-CER (d36:1), 808.7 -> 184.2 | 0.031298 | -0.13301* |  |
| SM (d42:3), 811.7 -> 184.2 | -0.03205 | -0.13933* |  |
| PC (38:3), 812.8 -> 184.2 | -0.0079363 | -0.14964* |  |
| PC (38:2); PI-CER (t34:0), 814.9 -> 184.2 | 0.018974 | -0.14197* |  |
| SM (d42:1), 815.5 -> 184.2 | -0.025513 | -0.13406* |  |
| PC (38:1), 816.5 -> 184.2 | -0.021776 | -0.13853* |  |
| **Method 5,** *m/z* |  |  |  |
| DG (30:2); PG (20:2); PA (24:0), 537.7 -> 283.2 | 0.14999* | 0.020438 |  |
| PC (18:0); PC (O-20:0); PE (22:0); LysoPE (22:0); PS (P-20:0); CER (d34:1), 538.3 -> 283.2 | 0.15058* | -0.003351 |  |
| DG (30:1); PG (20:1), 539.2 -> 283.2 | 0.010094 | -0.30029* |  |
| DG (30:1); PG (20:1), 539.3 -> 283.2 | -0.0099179 | -0.30281* |  |
| DG (30:1); PG (20:1), 539.4 -> 283.2 | -0.011485 | -0.30404* |  |
| DG (36:0), 630.2 -> 283.2 | 0.15011* | 0.040817 |  |
| * Loadings used for interpretation of PCA. | | | |


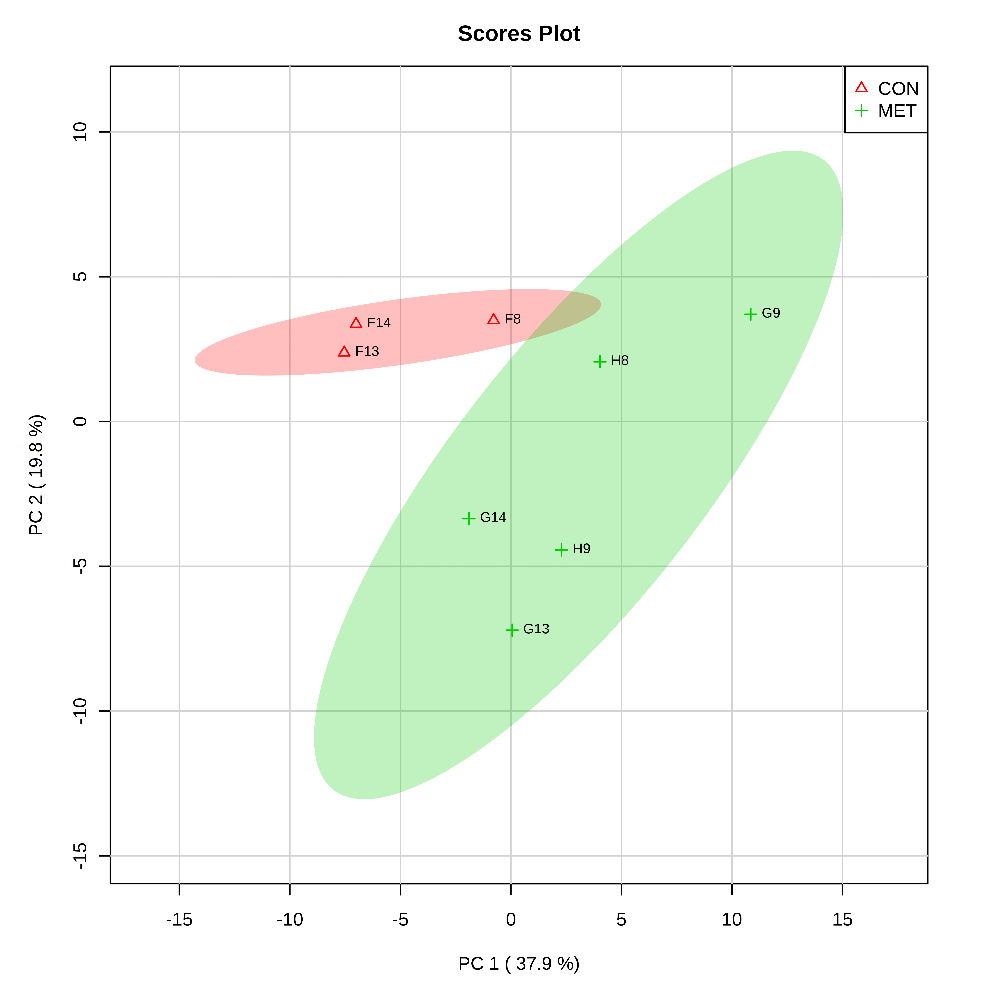

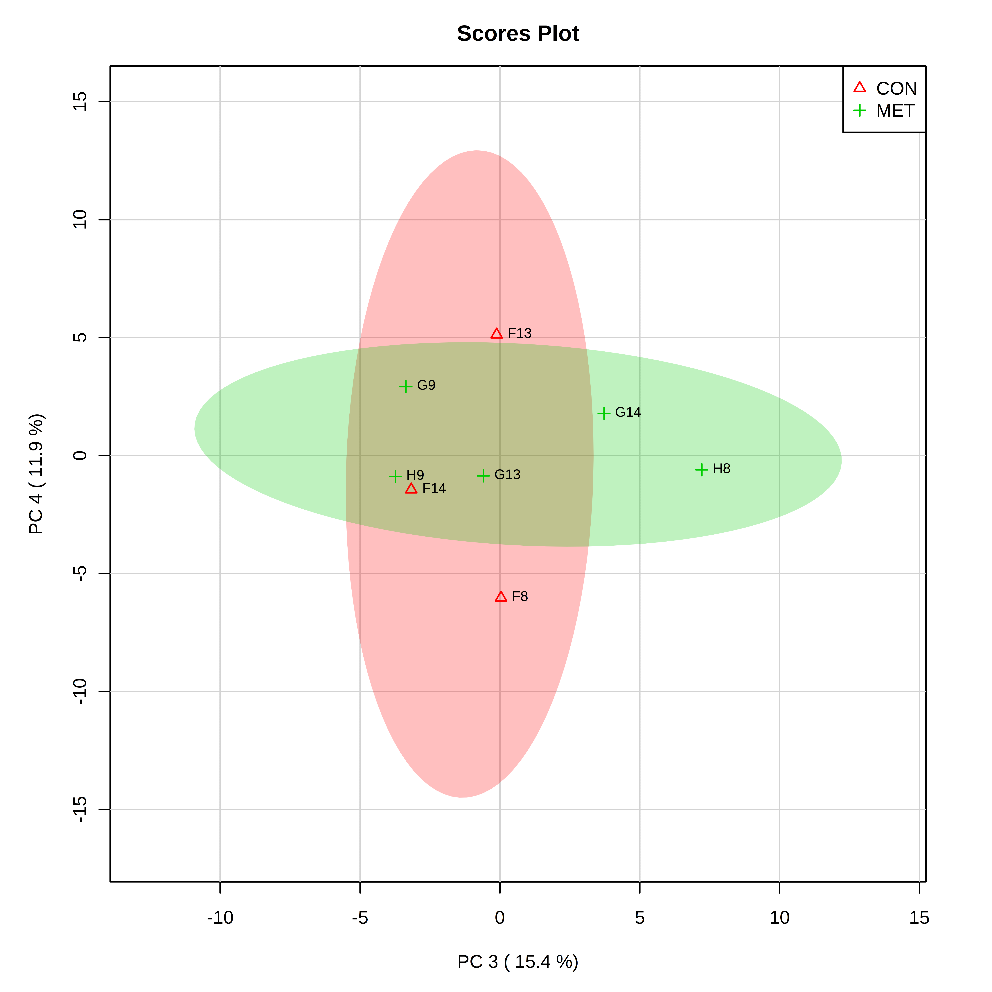


**Figure S1.** Principle component scores for lipid markers in embryos from cows (n = 8) fed rumen-protected methionine [MET +; n = 5 (G9, G13, G14, H8, H9)] or not [CON △; n = 3(F8, F13, and F14)].

**Figure S2.** Principle component scores for lipid markers in uterine biopsies obtained at 15 (A.), 30 (B.), and 73 (C.) DIM for method 1 from cows fed rumen-protected methionine [MET +; n = 11 (d15: 165, 8697, 8706, 8770, 8772; d30: 8697, 8706, 8770, 8772, 8781; d73: 847, 8390, 8550, 8697, 8706, 8768, 8772, 8781)] or not [CON △; n = 9 (d15: 8237, 8690, 8746, 8718, 28092; d30: 8237, 8348, 8690, 8718; d73: 8348, 8602, 8690, 8718, 8797)].


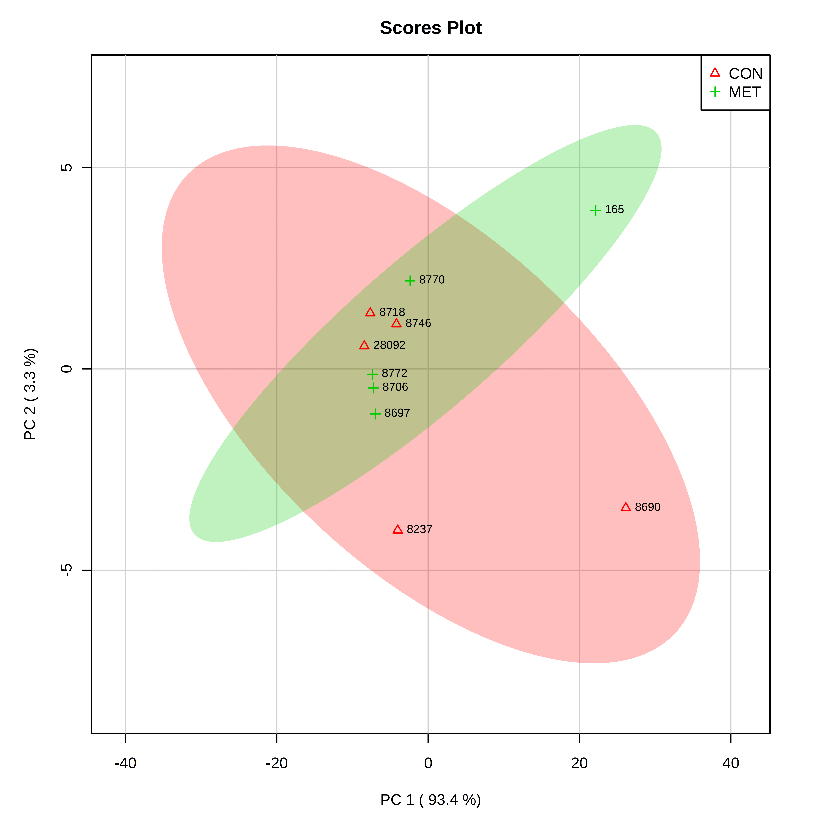

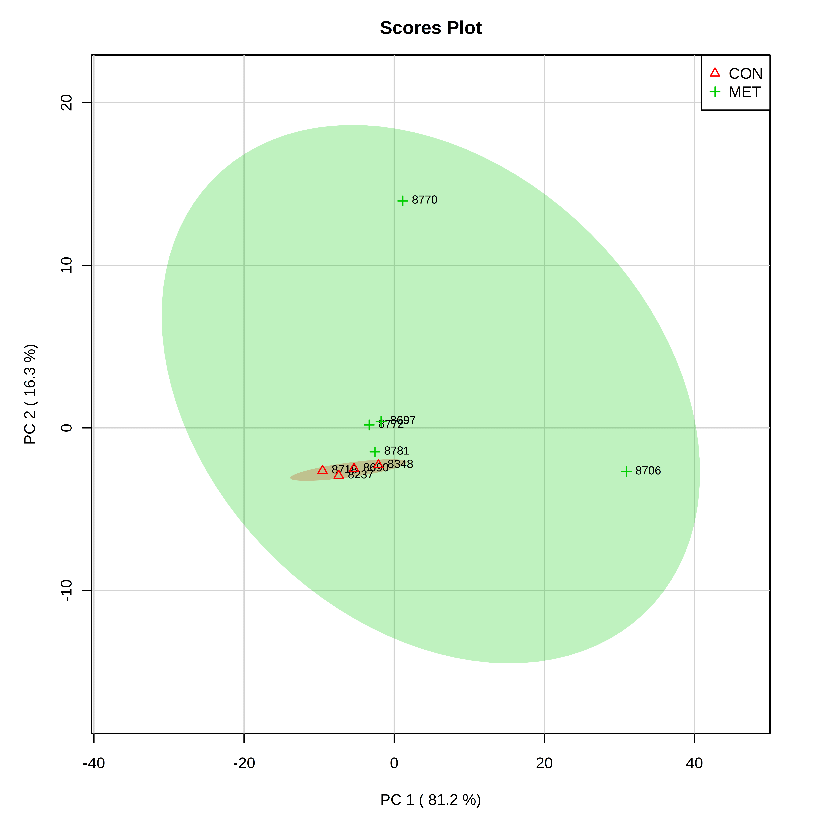

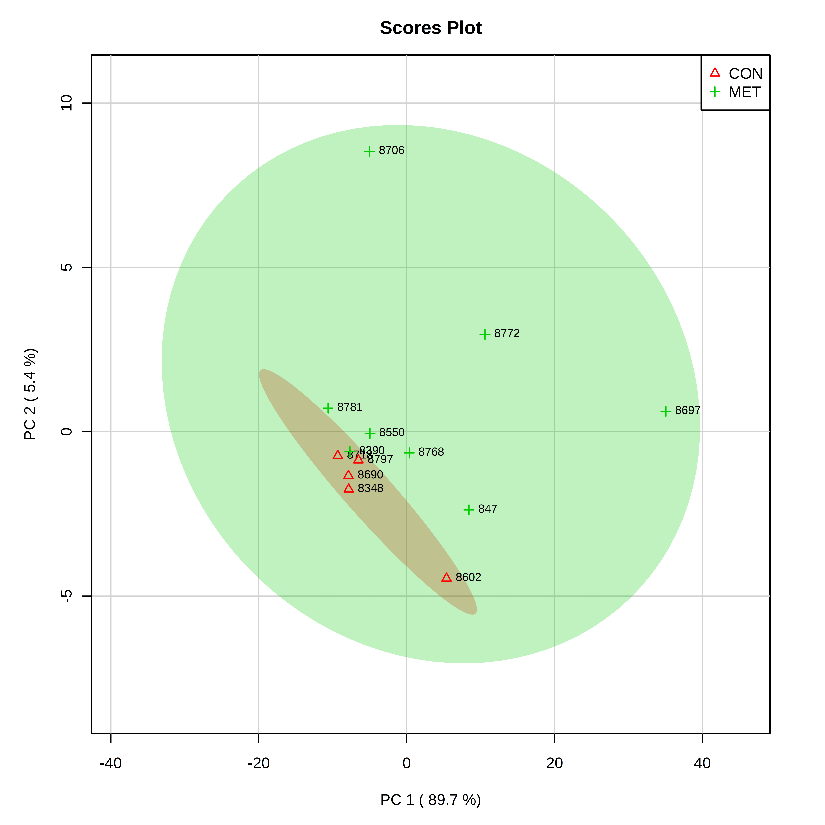


**A.**

**C.**

**B.**


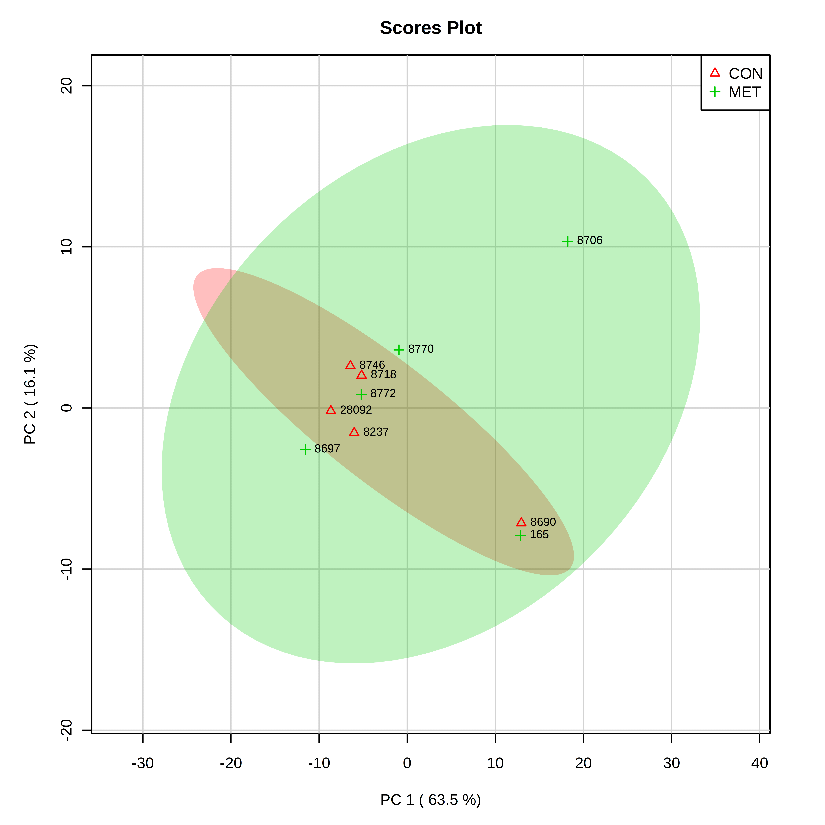

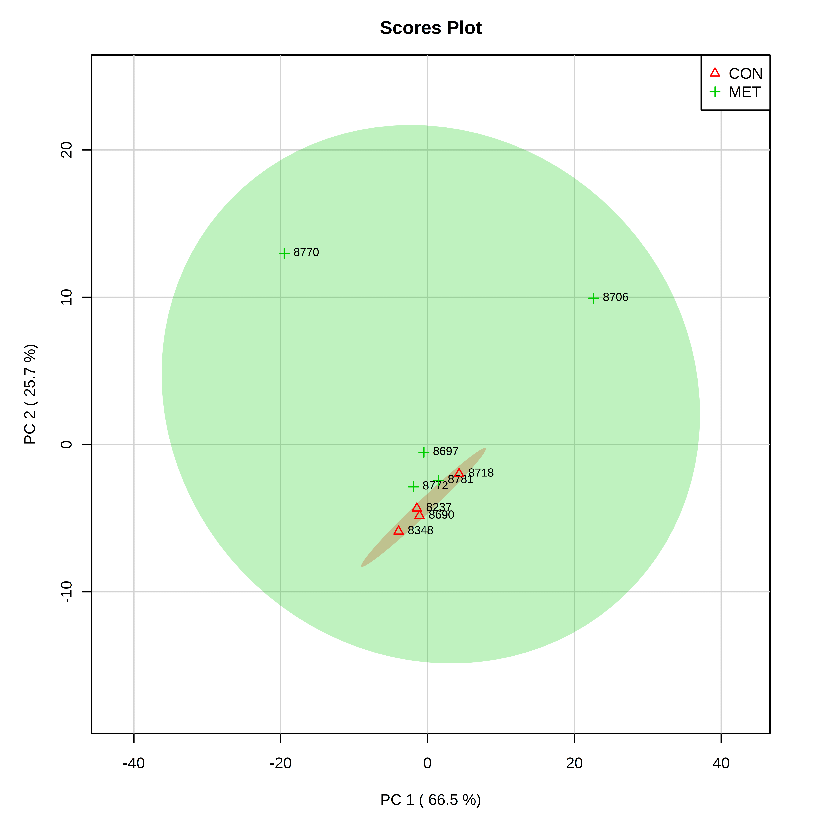

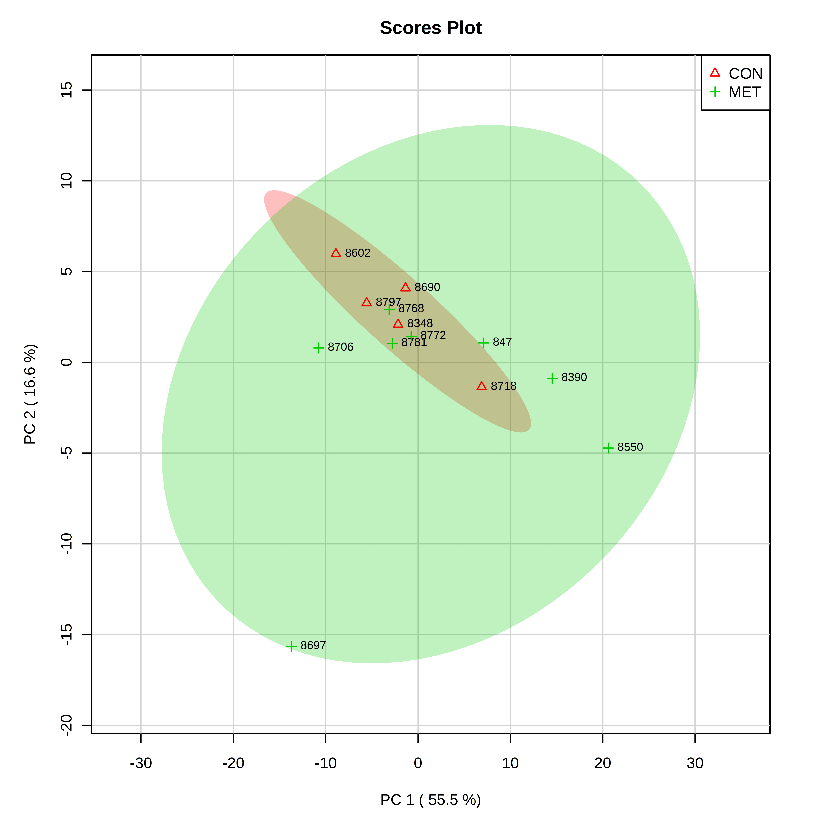

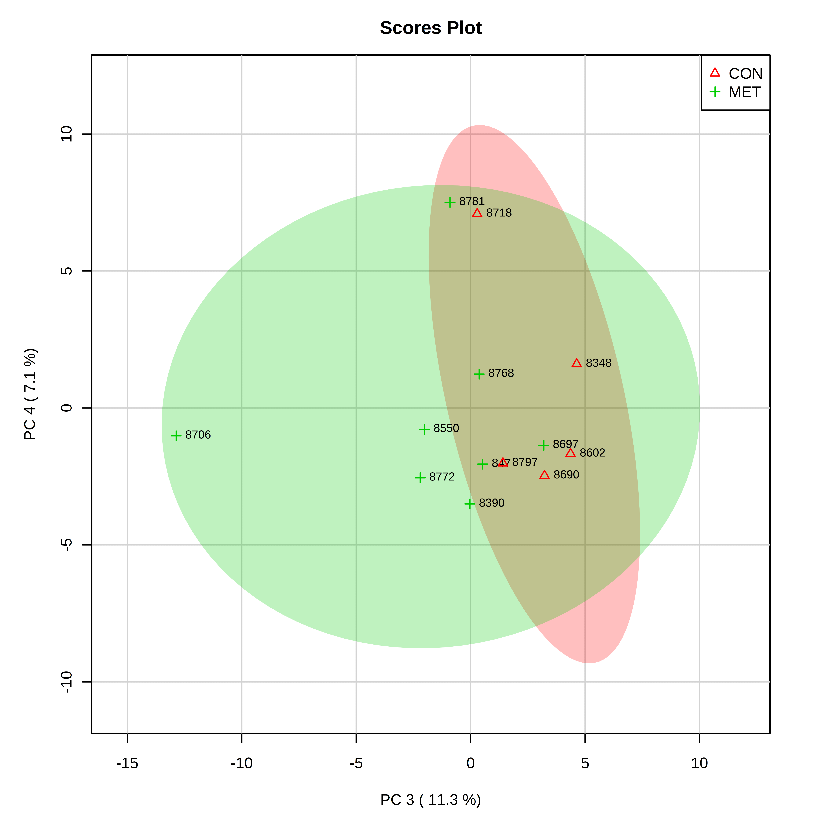


**B.**

**C.**

**D.**

**A.**

**Figure S3.** Principle component scores for lipid markers in uterine biopsies obtained at 15 (A.), 30 (B.), and 73 (C - D.) DIM for method 2 from cows fed rumen-protected methionine [MET +; n = 11 (d15: 165, 8697, 8706, 8770, 8772; d30: 8697, 8706, 8770, 8772, 8781; d73: 847, 8390, 8550, 8697, 8706, 8768, 8772, 8781)] or not [CON △; n = 9 (d15: 8237, 8690, 8746, 8718, 28092; d30: 8237, 8348, 8690, 8718; d73: 8348, 8602, 8690, 8718, 8797)].


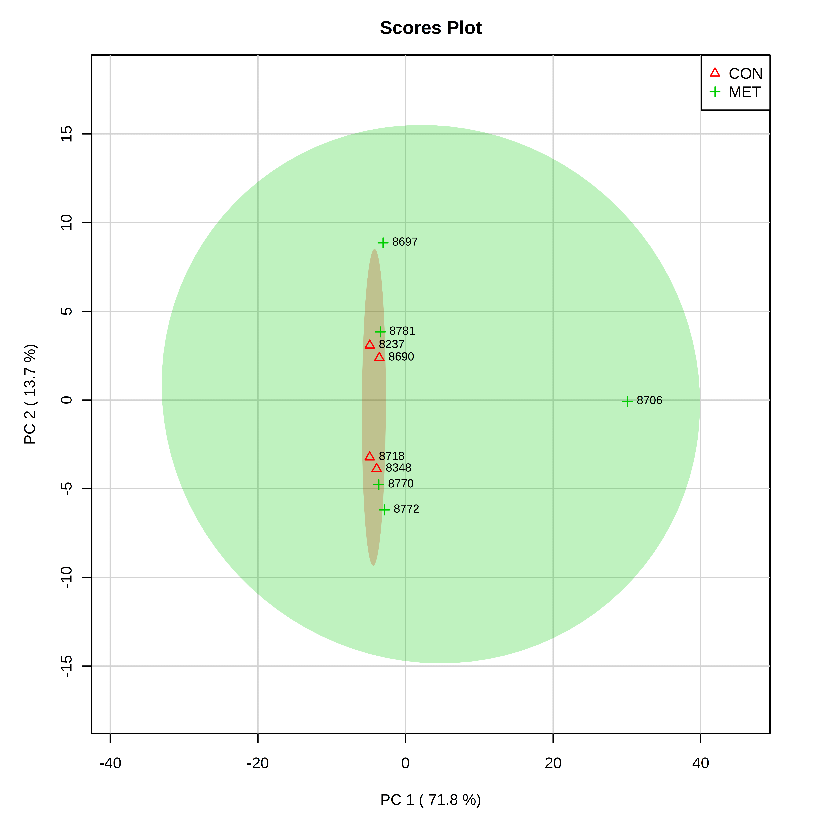

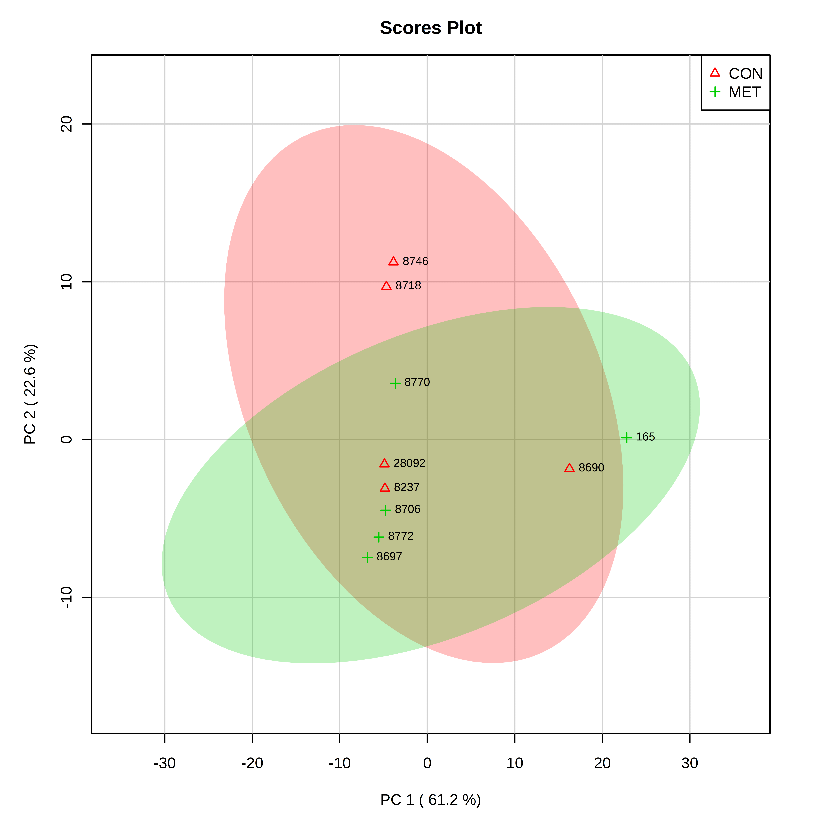

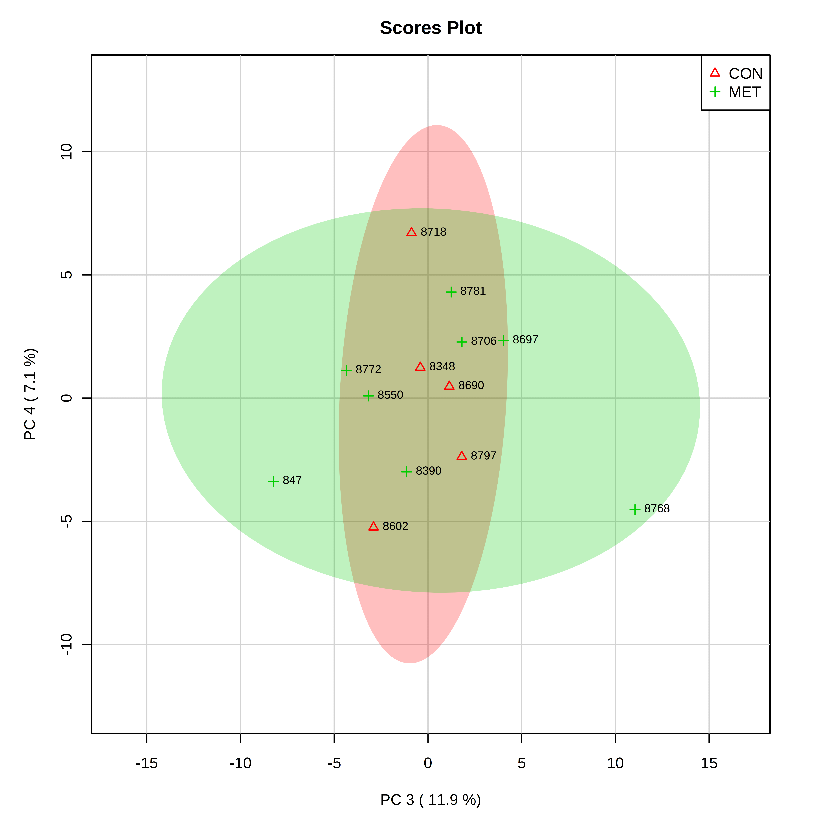

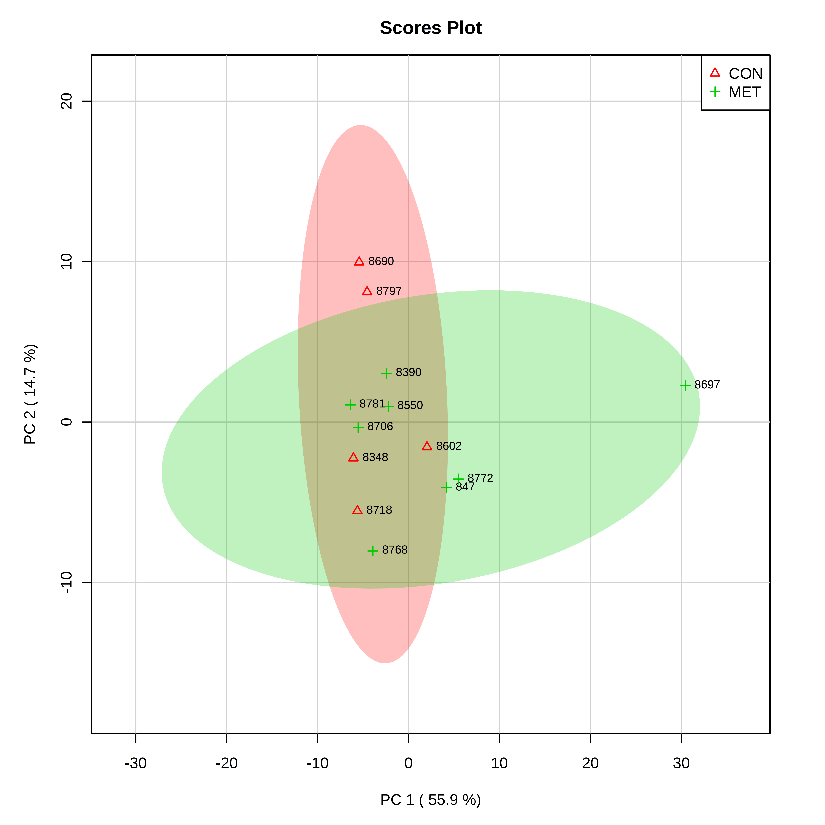


**C.**

**A.**

**B.**

**D.**

**Figure S4.** Principle component scores for lipid markers in uterine biopsies obtained at 15 (A.), 30 (B.), and 73 (C - D.) DIM for method 3 from cows fed rumen-protected methionine [MET +; n = 11 (d15: 165, 8697, 8706, 8770, 8772; d30: 8697, 8706, 8770, 8772, 8781; d73: 847, 8390, 8550, 8697, 8706, 8768, 8772, 8781)] or not [CON △; n = 9 (d15: 8237, 8690, 8746, 8718, 28092; d30: 8237, 8348, 8690, 8718; d73: 8348, 8602, 8690, 8718, 8797)].

**Figure S5.** Principle component scores for lipid markers in uterine biopsies obtained at 15 (A.), 30 (B.), and 73 (C.) DIM for method 4 from cows fed rumen-protected methionine [MET +; n = 11 (d15: 165, 8697, 8706, 8770, 8772; d30: 8697, 8706, 8770, 8772, 8781; d73: 847, 8390, 8550, 8697, 8706, 8768, 8772, 8781)] or not [CON △; n = 9 (d15: 8237, 8690, 8746, 8718, 28092; d30: 8237, 8348, 8690, 8718; d73: 8348, 8602, 8690, 8718, 8797)].


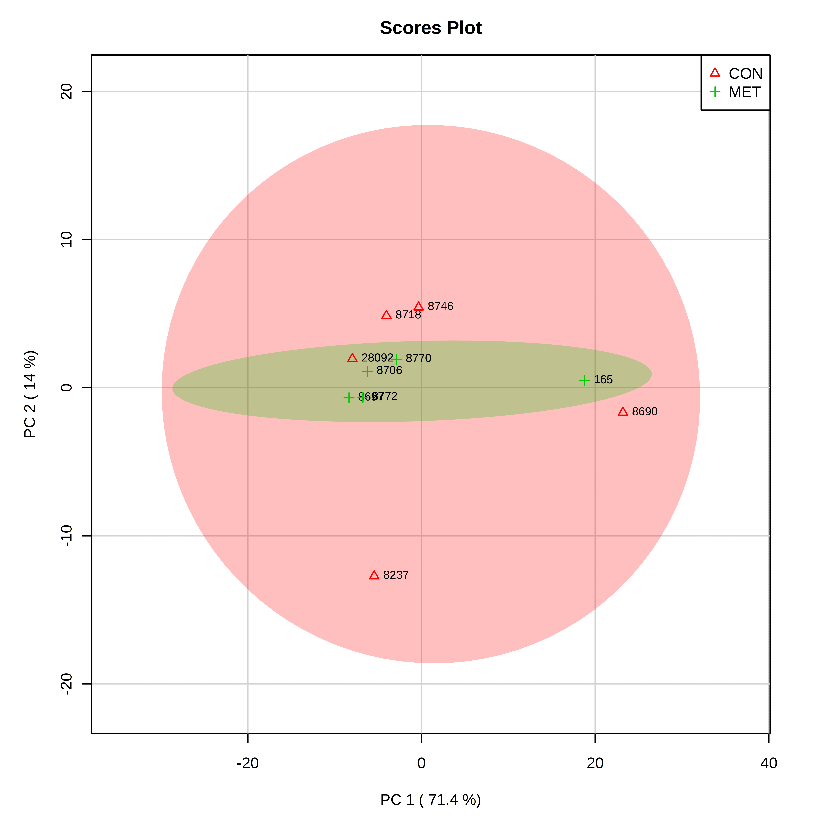

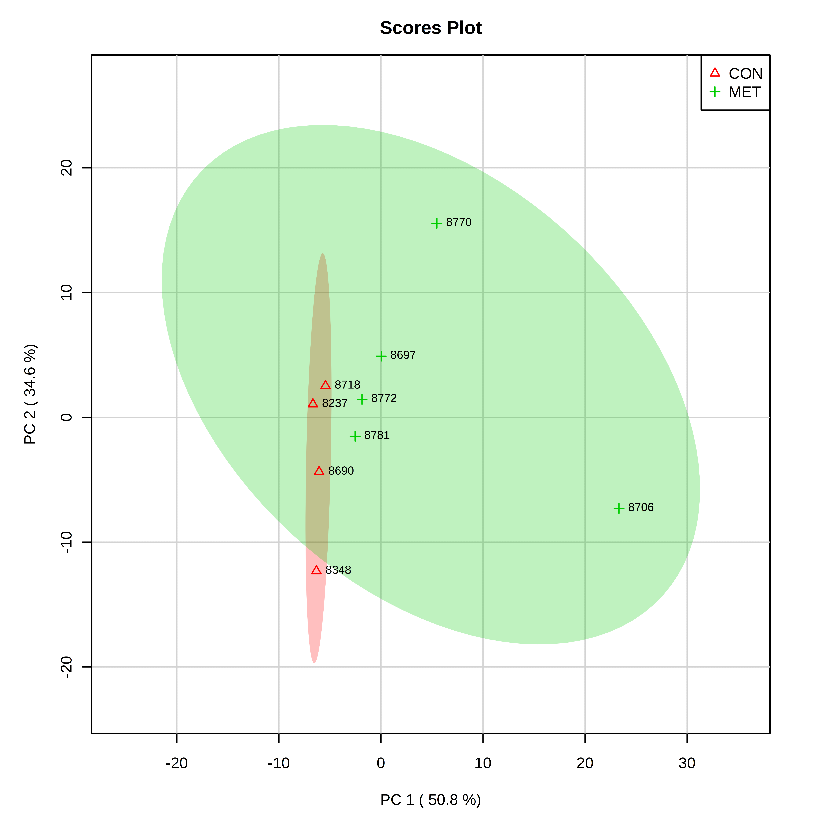

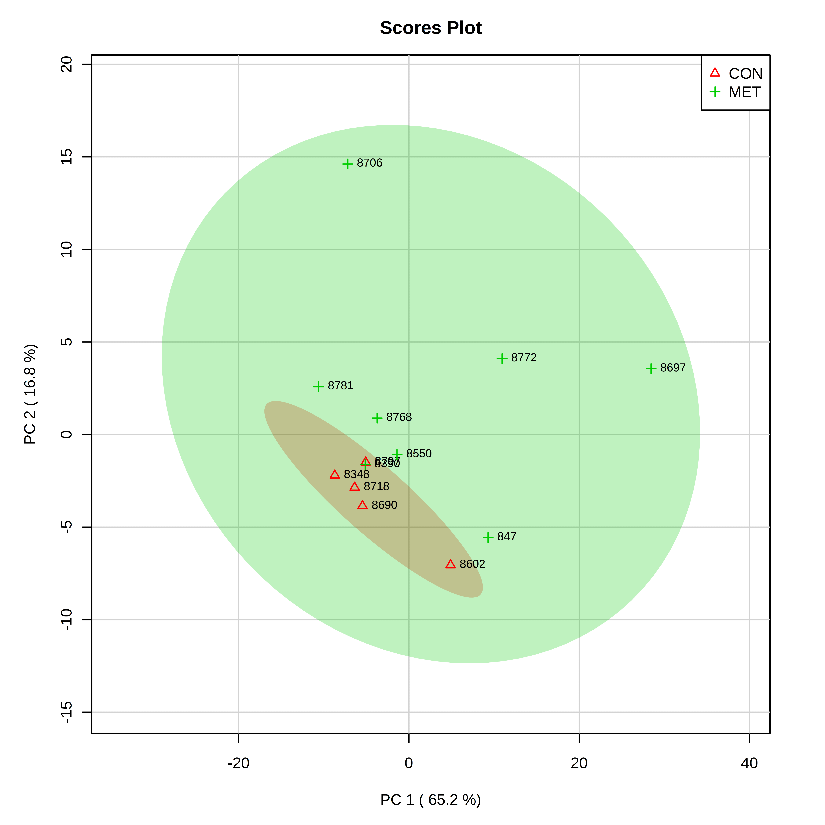


**B.**

**A.**

**C.**

**Figure S6.** Principle component scores for lipid markers in uterine biopsies obtained at 15 (A.), 30 (B.), and 73 (C.) DIM for method 5 from cows fed rumen-protected methionine [MET +; n = 11 (d15: 165, 8697, 8706, 8770, 8772; d30: 8697, 8706, 8770, 8772, 8781; d73: 847, 8390, 8550, 8697, 8706, 8768, 8772, 8781)] or not [CON △; n = 9 (d15: 8237, 8690, 8746, 8718, 28092; d30: 8237, 8348, 8690, 8718; d73: 8348, 8602, 8690, 8718, 8797)].


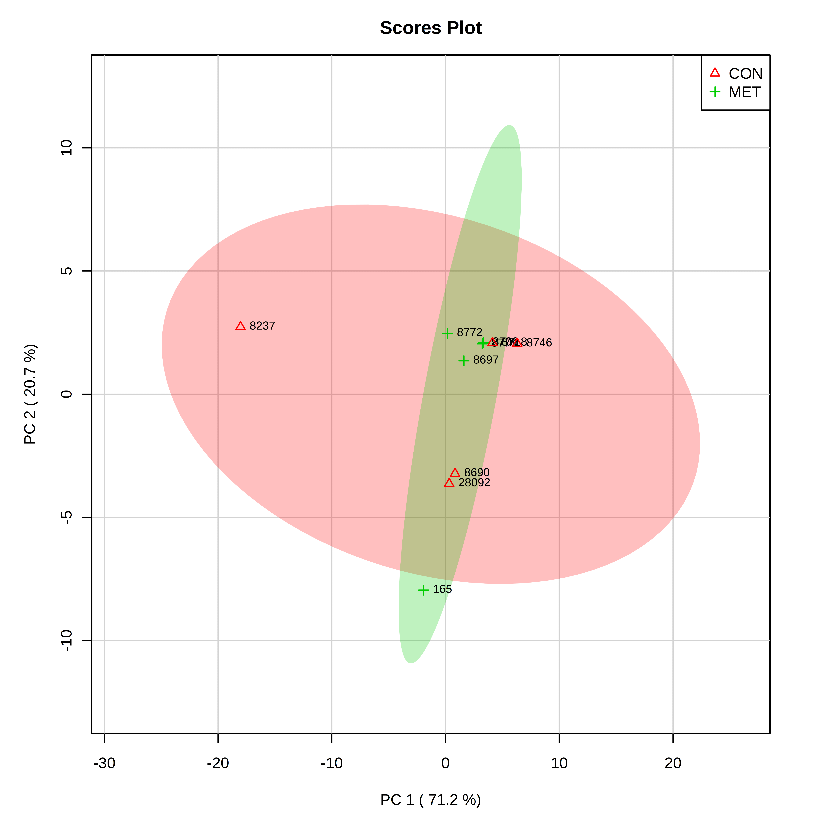

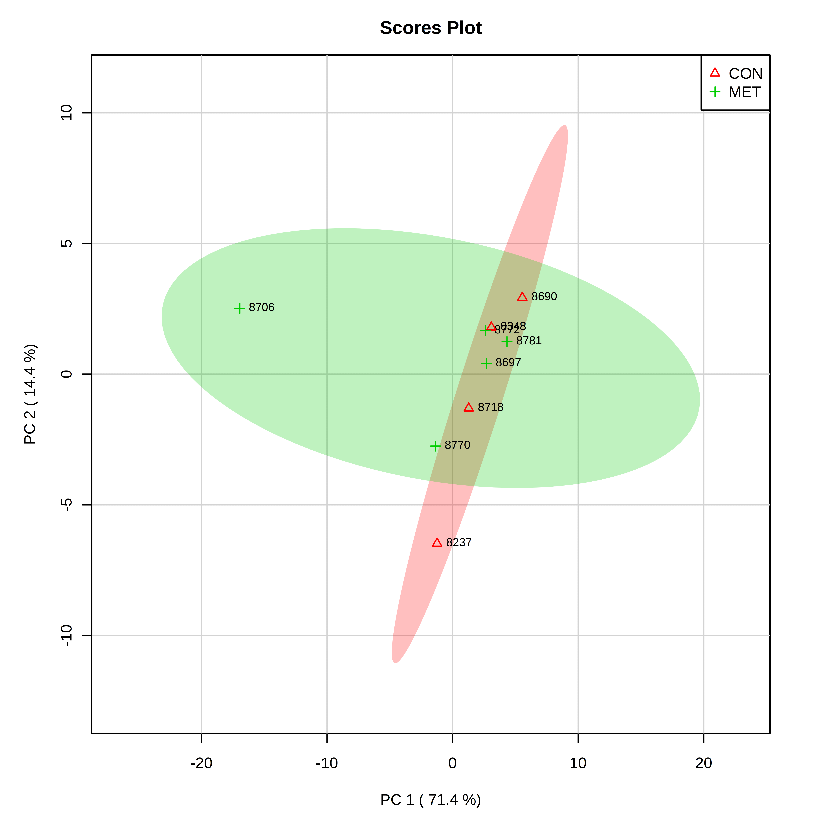

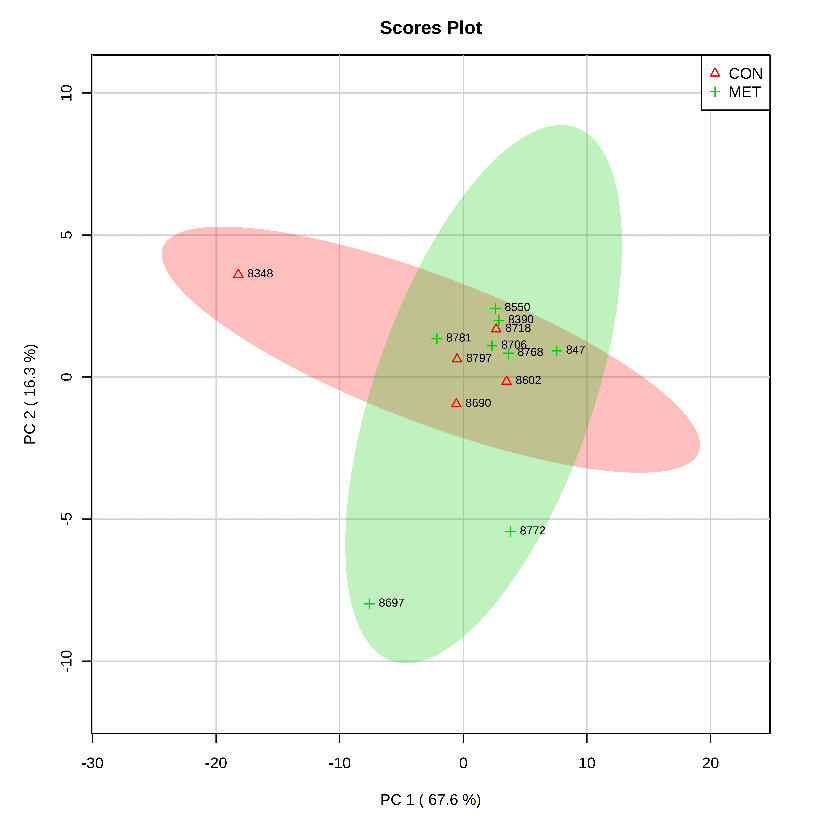


**C.**

**B.**

**A.**
